# A change of perspective in network centrality

**DOI:** 10.1038/s41598-018-33336-8

**Published:** 2018-10-15

**Authors:** Carla Sciarra, Guido Chiarotti, Francesco Laio, Luca Ridolfi

**Affiliations:** Department of Environmental, Land and Infrastructure Engineering, Politecnico di Torino, Corso Duca degli Abruzzi, 24, 10129 Torino, (IT) Italy

## Abstract

Typing “Yesterday” into the search-bar of your browser provides a long list of websites with, in top places, a link to a video by The Beatles. The order your browser shows its search results is a notable example of the use of network centrality. Centrality measures the importance of the nodes in a network and it plays a crucial role in several fields, ranging from sociology to engineering, and from biology to economics. Many centrality metrics are available. However, these measures are generally based on *ad hoc* assumptions, and there is no commonly accepted way to compare the effectiveness and reliability of different metrics. Here we propose a new perspective where centrality definition arises naturally from the most basic feature of a network, its adjacency matrix. Following this perspective, different centrality measures naturally emerge, including degree, eigenvector, and hub-authority centrality. Within this theoretical framework, the effectiveness of different metrics is evaluated and compared. Tests on a large set of networks show that the standard centrality metrics perform unsatisfactorily, highlighting intrinsic limitations for describing the centrality of nodes in complex networks. More informative *multi*-*component* centrality metrics are proposed as the natural extension of standard metrics.

## Introduction

Suppose a large number of individuals or entities interact in a network. A long-standing challenge is to rank these individuals for their relevance in the system, i.e., for the centrality of the nodes or agents in a network science jargon. In fact, centrality is referred to as a tool to quantify the importance of nodes in a network^1,2^. A first definition of this property dates back to the 50’s, when it was introduced to study the role of nodes in communication patterns^3,4^. During the following years, progress in social science provided several algorithms to evaluate nodes’ centrality. These methods were typically obtained through case-specific considerations about the functioning of social networks, mainly based on reasonings about how information spreads across people in a group^3^, and afterwards they were extended to other networks. Examples include the degree centrality^5,6^, the Katz centrality^7^, the eigenvector centrality^8^, the betweeness^6,9^ and the closeness centrality^6^, the PageRank^10^, the subgraph centrality^11^, and the total communicability^12^. Each metric defines node’s centrality on the basis of some topological features of the considered node, such as the number of its connections, the connections of its neighbours, the number of walks and paths going across the node, etc. All the metrics hence provide different answers to the question “*what does it mean to be central in a network*?” (see, e.g.^13–15^ for a literature review on centrality indexes and definitions). Due to the growing number of problems framed in network science, answering to the question about the meaning of node centrality is crucial for many scientific and technical field, ranging from epidemiology^16–18^ to economics^19–22^, from sociology^23^ to engineering^24,25^ and neuro-sciences^26,27^.

Several different measures of node centrality exist, each one with its own merits and peculiarities. The formulation of centrality metrics, in fact, typically descends from *ad hoc* assumptions, where a node is said to be central if it has some specific features which testify its relevance in the network, with possible risks of circular reasoning. For example, one may assume a node is more central if it has many connections with other nodes, which leads to the degree centrality as the natural measure. However, one may argue that nodes are not all equivalent, and that a weighted version of the degree of the nodes should be adopted, where the weight is the centrality itself: this leads to the eigenvector centrality as the adequate metric. Both these measures have a solid intuitive background. Nevertheless, one is left without the possibility of comparing the reliability of different measures of centrality, and therefore, of choosing which is the most effective metric – and resulting node ranking – for the specific problem at hand.

Aiming at providing a more grounded deductive framework, we propose to tackle the centrality problem as a matrix-estimation exercise. The proposed approach allows one (i) to deduce a hierarchy of metrics, (ii) to recast classical centrality measures (degree, eigenvector, Katz, hub-authority centrality) within a single theoretical scheme, (iii) to compare different centrality measures by evaluating their performances in terms of their capability to reproduce the network topology, and (iv) to extend the notion of centrality to a multi-component setting, still maintaining the possibility to use centrality to rank the nodes.

This new perspective on centrality is general and can be applied to any network: undirected/directed, unweighted/weighted, and monopartite/bipartite networks.

## The New Perspective: Undirected, Unweighted Networks

Let *G* be an undirected, unweighted graph, with *N* nodes and *E* edges. *G* is mathematically described by the symmetric adjacency matrix **A**, whose *ij*-th element is 1 if *i* and *j* share an edge, zero otherwise^2^. Let $\hat{{\bf{A}}}$ be an estimator of the adjacency matrix. We expect a good estimator has larger ${\hat{A}}_{ij}$ values when *i* and *j* are connected (i.e., *A*_*ij*_ = 1), and lower values otherwise (i.e., when *A*_*ij*_ = 0). Our key idea is that the estimator of the generic element *A*_*ij*_ should depend on some emerging property *x*_*i*_ of the node *i* and *x*_*j*_ of the node *j* (with *i*, *j* = 1:*N*) representing the topological importance of each node, i.e. its centrality. In formulas, ${\hat{A}}_{ij}=f({x}_{i},{x}_{j})$ where *f* is an increasing function of both its arguments, since ${\hat{A}}_{ij}$ should increase when the nodes *i* and *j* are more “central” in the network. Due to the symmetry of the matrix **A**, the arguments of *f* should also be exchangeable (i.e., *f*(*x*_*i*_, *x*_*j*_) = *f*(*x*_*j*_, *x*_*i*_)). Notice that the estimation process projects the information from *N*^2^ to *N* as we are estimating a *N* × *N* matrix using the *N* values of nodes’ centrality *x*_*i*_. By definition, estimation is non exact, and ${A}_{ij}\ne {\hat{A}}_{ij}$. We suppose here that the error *ε*_*ij*_ related to the estimation is in additive form, namely

1 $${A}_{ij}={\hat{A}}_{ij}+{\varepsilon }_{ij}=f({x}_{i},{x}_{j})+{\varepsilon }_{ij}.$$

Under this perspective, the centrality measures can be obtained on sound statistical bases, as they arise as the result of a standard estimation problem. Different constraints about the error structure can be considered. The most classical approach – least squares estimation – entails minimising the sum of the squared errors, i.e.

2 $$SE({x}_{1},{x}_{2},\ldots ,{x}_{N})=\sum _{i}\,\sum _{j}\,{\varepsilon }_{ij}^{2}=\sum _{i}\,\sum _{j}\,{({A}_{ij}-f({x}_{i},{x}_{j}))}^{2}.$$

By minimising this quantity with respect to *x*_*i*_, i.e., solving the equation (see SI, Sect. 1)

3 $$\frac{{\rm{\partial }}SE}{{\rm{\partial }}{x}_{i}}=4\,\sum _{j}\,[{A}_{ij}-f({x}_{i},{x}_{j})]\cdot {\frac{{\rm{\partial }}f({z}_{m},{x}_{j})}{{\rm{\partial }}{z}_{m}}|}_{{z}_{m}={x}_{i}}=0,$$

(where *z*_*m*_ is a bound variable), a set of *N* equations is obtained, which allows one to estimate the centrality value for all nodes. In Eq. (3), the bound variable *z*_*m*_ allows one to formalize more concisely the mathematics behind the rationale (see SI, Sect. 1). Notice that the framework can be extended to consider the error term in Eq. (1) in multiplicative form, and/or to consider a node-wise unbiased constraint instead of minimising *SE*.

Within this statistical framework, the answer to the question “*what does it mean to be central in a network*?” is given through the analysis of the importance of the nodes in the estimation of *A*_*ij*_: a node *i* is more central than a node *j* if the effect of its property *x*_*i*_ on the minimisation of *SE* is larger i.e., if it is more “useful” for estimating **A**. Put it another way, the node *i* is more important than the node *j* if, when removing its property from the estimation of *A*_*ij*_, the change in *SE* recorded is higher than the one provoked by the exclusion of other nodes’ property *x*_*j*_. In order to account for this effect, we borrow the concept of the *unique contribution* from the theory of commonality analysis^28,29^. The unique contribution is a quantitative measure of the effect a single variable has in the estimation procedure^30^. We define the unique contribution of the node *i* as the gain in the coefficient of determination *R*^2^ induced by considering *x*_*i*_ in the estimation procedure. In formulas

4 $$U{C}_{i}={R}_{N}^{2}-{R}_{N\backslash i}^{2}=\frac{S{E}_{N\backslash i}-S{E}_{N}}{TSS},$$

where ${R}^{2}=1-\frac{SE}{TSS}$, with *SE* as in Eq. (2), and $TSS={\sum }_{i}\,{\sum }_{j}\,{({A}_{ij}-\bar{A})}^{2}$, with $\bar{A}={\sum }_{i}\,{\sum }_{j}\,{A}_{ij}/{N}^{2}$ (see SI, Sect. 1.1 for details). The subscripts *N* and *N*\*i* in Eq. (4) refer to the case when all the *x*_*i*_ values are considered in the estimation (subscript *N*), or to the case when the *i*-th property is excluded (subscript *N*\*i*). If the *UC* of node *i* is larger compared with the one obtained for node *j*, excluding *x*_*i*_ from the estimation produces a larger drop in our capacity to estimate the adjacency matrix (i.e., a larger drop in *R*^2^). As a consequence, the larger is *UC*_*i*_, the most relevant (or central) the node is for reconstructing the adjacency matrix with a limited amount of information (i.e., the *N* centrality values). This allows one to perform a ranking of the network nodes for their capacity to contribute to the network estimation. According to the commonality analysis, the unique contribution should be computed eliminating the *i*-th node and repeating the estimation procedure with (*N* − 1) variables, in order to compute the determination coefficient ${R}_{N\backslash i}^{2}$. However, this approach would entail repeating the estimation for (*N* + 1) times, a potentially cumbersome effort in large networks. To bypass this difficulty, in this work we set a baseline scenario in which the *i*-th node is not formally excluded from the estimation, but the computation of the *UC*_*i*_ is performed setting to zero the centrality value *x*_*i*_ in the estimation procedure (see SI, Sect. 1.1). This also allows one to keep the results in analytical form. As will be clear in the following, the assumption *x*_*i*_ = 0 corresponds to assume a node with the lowest possible centrality value, since the centrality values are positive-valued. This assumption does not necessarily entail that the estimated link between two nodes *i* and *j* does not exist.

Different definitions of the function *f* in Eq. (1) allow one to obtain different centrality metrics. Some noteworthy examples are described in Table 1. The degree centrality, the eigenvector centrality^8^ and the Katz centrality^7^ are obtained by adopting very simple link-estimation functions. Recasting these centrality metrics into this new framework allows us to compare their performances, in terms of their ability to predict the adjacency matrix. New metrics can also be easily obtained, by adopting the estimator function *f* which is the most suitable to represent the matrix-estimation problem at hand.

**Table 1:** Examples of the estimator functions *f* to be set in Eq. (1) to obtain some commonly-used centrality measures.

Undirected networks			
Estimator function *f*	Centrality of node *i*	Unique contribution of node *i*	Corresponding metric
${f}_{1}=\tfrac{{K}_{tot}}{N}({x}_{i}+{x}_{j}-\tfrac{1}{N})$	${x}_{i}=\tfrac{{k}_{i}}{{K}_{tot}}$	$U{C}_{i}=\tfrac{2(N+1){k}_{i}^{2}}{{N}^{2}\,TSS}$	Degree centrality
*f*_2_ = *γx*_*i*_*x*_*j*_	${x}_{i}=\tfrac{1}{\gamma }\,{\sum }_{j}\,{A}_{ij}{x}_{j}$	$U{C}_{i}=\tfrac{\gamma {x}_{i}^{2}}{TSS}(\gamma {x}_{i}^{2}+2\gamma )$	Eigenvector centrality
*f*_3_ = *γx*_*i*_*x*_*j*_ + *B*	${x}_{i}=\tfrac{{\sum }_{j}\,{A}_{ij}{x}_{j}}{\gamma \,{\sum }_{j}\,{x}_{j}^{2}}+\tfrac{B\,{\sum }_{j}\,{x}_{j}}{\gamma \,{\sum }_{j}\,{x}_{j}^{2}}$	$U{C}_{i}=\tfrac{\gamma {x}_{i}^{2}}{TSS}(\gamma {x}_{i}^{2}-2B+2\gamma \,{\sum }_{j}\,{x}_{j}^{2})$	Katz centrality

The unique contribution, which is here used to rank nodes for their centrality, is also reported. In the formulas, ${K}_{tot}={\sum }_{i}\,{\sum }_{j}\,{A}_{ij}$ is the total degree of the network; *N* is the number of nodes; ${k}_{i}={\sum }_{j}\,{A}_{ij}$ is the degree of the node *i*; *γ* and *B* are two parameters whose values change according to the estimator function. In case of *f*_2_, *γ* equals the largest eigenvalue of **A**. In case of *f*_3_, $\gamma =1/\alpha \,{\sum }_{j}\,{x}_{j}^{2}$ and $B=-\,1/{\sum }_{j}\,{x}_{j}$, where *α* is the *attenuation factor* of the Katz centrality. *TSS* is defined in the text. Further details are given in SI, Sect. 1.

Some readers may recognise a formal resemblance between our *f*(*x*_*i*_, *x*_*j*_) and the function used to attribute a probability of link activation based on the nodes’ *fitness*^31,32^. However, the perspective is reversed here. In fact we are not aiming to generate a suitable network structure with a given node property distribution, but we are estimating the nodes’ properties that best represent a given adjacency matrix.

## Extending The New Perspective

A natural extension of the *one*-*component* estimators (Table 1) is to move toward more informative *multi*-*component* metrics of nodes’ centrality. The multi-component centrality considers more facets of the network, by describing the role of network’s nodes through more than one scalar property. In formulas ${\hat{A}}_{ij}=f({{\bf{x}}}_{i},{{\bf{x}}}_{j})$, where ${{\bf{x}}}_{i}=[{x}_{i,1},\ldots ,{x}_{i,s}]$ is an *s*-dimensional vector embedding the *s* properties of the node that should be considered for evaluating its importance (for *s* = 1 the one-component metrics are recovered).

By taking the function *f*_2_ in Table 1 as the starting point for our reasoning, a possible design of the multidimensional estimator is obtained,

5 $${\hat{A}}_{ij}(s)={\gamma }_{1}{x}_{i,1}{x}_{j,1}+\cdots +{\gamma }_{k}\,{x}_{i,k}\,{x}_{j,k}+\cdots +{\gamma }_{s}{x}_{i,s}{x}_{j,s}.$$

A multivariate extension of the function *f*_1_ in Table 1 is useless, because in the additive form the contribution carried by different variables $({x}_{i,1},\mathrm{..},{x}_{i,s})$ cancels out if one refers to a single variable, *ξ*_*i*_, which is a linear combination of the different components. In other words, the components beyond the first one cannot bring any additional information into the estimation exercise. An extension of *f*_3_ would instead simply imply to add a constant value to Eq. (5).

Using Eq. (5), the estimation process projects *N*^2^ (i.e. the number of entries of the adjacency matrix) data to $s\cdot N$, which is the number of independent variables used in the estimation.

One may recognise that the formal structure of $\hat{{\bf{A}}}$ in Eq. (5) corresponds to the *s*-*order low*-*rank approximation* of the matrix **A**^33^. Under a least squares constraint, and the assumption of orthogonality between the *s* vectors x_*k*_, one obtains that *γ*_*k*_ is the *k*-th eigenvalue of the adjacency matrix and ${{\bf{x}}}_{k}=[{x}_{1,k},\ldots ,{x}_{N,k}]$ is its corresponding eigenvector (see SI, Sect. 1.5). Sorting the eigenvalues in descending order according to their absolute value, eigenvectors of increasing order bring a monotonically decreasing amount of information. This solution corresponds to the *Singular Value Decomposition* (SVD)^33^ of the original matrix, truncated at the order *s* (see SI, Sect. 1.5). The choice of the *s* value therefore entails finding a good balance between the necessity to accurately describe the adjacency matrix and the willingness to have a parsimonious representation of a complex system. Different strategies can be pursued, also borrowing from the wide literature pertaining with the similar problem of deciding where to arrest the eigenvalue decomposition or the SVD (see, e.g.^34^ for a review). For example, one may choose the *s* value corresponding to the first gap in the eigenspectrum of the adjacency matrix (see, e.g.^35^). Alternatively, one may stop the expansion in Eq. (5) when the explained variance reaches a predefined amount of the total variance of **A**. This would entail that the remaining amount of variance is attributed to noise.

The unique contribution of the *i*-th node, and hence its centrality value, when the expansion is arrested to *s* is obtained by setting *x*_*i*,*k*_ = 0, for *k* = 1:*s*. Interpreting the multi-component extension as a vector, this assumption corresponds to taking the vector module down to zero, which again entail minimising the node centrality as in the 1-dimensional case. This provides (see SI, Sect. 1.5.1)

6 $$U{C}_{i}(s)=\frac{1}{TSS}\,[{(\sum _{k=1}^{s}{\gamma }_{k}{x}_{i,k}^{2})}^{2}+2\,\sum _{k=1}^{s}\,{\gamma }_{k}^{2}{x}_{i,k}^{2}].$$

The *x*_*i*,*k*_ values in Eq. (6) appear in squared form. As a consequence, the sign of *x*_*i*,*k*_ does not affect the *UC*_*i*_ value.

It is clear that, by considering additional dimensions beyond the first, the node centrality ranking may significantly change, revealing node features which were hidden by the one-dimensional assumption. In fact, information on the structure and clustering of the network is contained in the eigenvectors beyond the first one (for more information see, e.g.^35–37^). In the case *s* = *N*, through the *UC* one recovers the same ranking given by the degree centrality. In fact, in this case the approximated matrix equals the adjacency matrix, i.e., $\hat{{\bf{A}}}={\bf{A}}$ and the errors are zero. In contrast, since the *i*-th row and column of $\hat{{\bf{A}}}$ are zero when excluding the *i*-th node from the estimation, ${R}_{N\backslash i}^{2}$ turns out to be proportional to the squared degree of node *i*, ${k}_{i}^{2}$. Therefore, when considered under the perspective of the unique contribution, the expansion with *s* = *N* copies the same information of the node degree, in terms of the obtained nodes’ ranking. It may be useful to note that the multi-component estimation of centrality, and the subsequent ranking given through the *UC*, entail a two-steps shrinkage of information. Firstly, the estimation projects data from *N*^2^ to $s\cdot N$, and secondly the ranking projects from $s\cdot N$ to *N*. Therefore, the multi-component centrality acts as an additional pier for the bridge from *N*^2^ to *N*, a pier which can be essential to pose the centrality estimation problem on more solid grounds. Clearly, both cases *s* = 1 and *s* = *N* correspond to limit situations when the additional pier is not in between *N*^2^ and *N*, but it is on one of the two sides; in fact, in these situations one recovers the eigenvector centrality (*s* = 1) and the degree centrality (*s* = *N*).

## The New Perspective: Other Network Classes

### Directed, unweighted networks

In directed, unweighted networks, edges are directed and the elements *A*_*ij*_ of the adjacency matrix **A** are 1 if the edge points from *i* to *j*, and zero otherwise. The adjacency matrix is generally asymmetric^2^ (notice that we here consider *i* pointing to *j* i.e., the outgoing edges of the node *i* are described onto the row *i* of the matrix **A**). In this kind of networks, nodes can be characterised by two properties, one concerning with the *outgoing* centrality of the node, ${x}_{i}^{out}$, and the other concerning with the *incoming* centrality, ${x}_{i}^{in}$. The estimator ${\hat{A}}_{ij}$ should depend on the outgoing centrality of node *i* and on the incoming centrality of node *j*, namely ${\hat{A}}_{ij}=f({x}_{i}^{out},{x}_{j}^{in})$. Examples of the *out* and *in* centrality of the nodes recovered in this statistical framework are the degree and the hub-authority centrality^38^ (see Table 2, details in SI, Sect. 2). Within this framework, the unique contribution can also be used to produce an overall ranking of network’s nodes, combining both the *out* and *in* centrality of the nodes (see SI, Sect. 2).

**Table 2:** Estimator functions used for directed networks.

Directed networks			
Estimator function *f*	Out, in and total centrality of node *i*	Out, in and total unique contribution of node *i*	Corresponding metric
${f}_{1}=\tfrac{{K}_{tot}}{N}({x}_{i}^{out}+{x}_{j}^{in}-\tfrac{1}{N})$	${x}_{i}^{out}=\tfrac{{k}_{i}^{out}}{{K}_{tot}}$ ${x}_{j}^{in}=\tfrac{{k}_{j}^{in}}{{K}_{tot}}$ ${x}_{j}^{in}=\tfrac{{k}_{j}^{in}}{{K}_{tot}}$	$U{C}_{i}^{out}=\tfrac{{({k}_{i}^{out})}^{2}}{N\,TSS}$, $U{C}_{i}^{in}=\tfrac{{({k}_{i}^{in})}^{2}}{N\,TSS}$ $U{C}_{i}^{tot}=\tfrac{1}{TSS}(\tfrac{{({k}_{i}^{out})}^{2}+{({k}_{i}^{in})}^{2}}{N}+\tfrac{2{k}_{i}^{out}{k}_{i}^{in}}{{N}^{2}})$	Degree centrality
${f}_{2}=\gamma {x}_{i}^{out}{x}_{j}^{in}$	$$\{\begin{array}{rcl}{x}_{i}^{out} & = & \tfrac{1}{\gamma }\,\sum _{j}\,{A}_{ij}{x}_{j}^{in}\\ {x}_{j}^{in} & = & \tfrac{1}{\gamma }\,\sum _{i}\,{A}_{ij}{x}_{i}^{out}\end{array}$$	$U{C}_{i}^{out}=\tfrac{{(\gamma {x}_{i}^{out})}^{2}}{TSS}$, $U{C}_{i}^{in}=\tfrac{{(\gamma {x}_{i}^{in})}^{2}}{TSS}$ $U{C}_{i}^{tot}=\tfrac{1}{TSS}[{\gamma }^{2}({({x}_{i}^{out})}^{2}+{({x}_{i}^{in})}^{2})+{(\gamma {x}_{i}^{out}{x}_{i}^{in})}^{2}]$	Hub-authority centrality

In the formulas, *K*_*tot*_ is the total degree of the network; *N* is the number of nodes; ${k}_{i}^{out}$ and ${k}_{i}^{in}$ are the *out* degree and *in* degree of the node *i*; *γ* is a parameter whose value equals the principal singular value *σ*_1_ of **A**. *TSS* is defined in the text. The equations for the unique contribution are reported for the cases when outgoing and incoming properties of the node are separately considered (superscripts *out* and *in*), or for the case when they are considered together (superscript *tot*). Further details are given in SI, Sect. 2.

The expansion to *multi*-*component* centrality and estimator, is a function of the *s*-dimensional vectors of the nodes’ properties ${{\bf{x}}}_{i}^{out}$ and ${{\bf{x}}}_{j}^{in}$, namely

7 $${\hat{A}}_{ij}(s)={\gamma }_{1}{x}_{i,1}^{out}{x}_{j,1}^{in}+\cdots +{\gamma }_{k}\,{x}_{i,k}^{out}{x}_{j,k}^{in}+\cdots +{\gamma }_{s}{x}_{i,s}^{out}{x}_{j,s}^{in}.$$

Eq. (7) coincides with the Singular Value Decomposition (SVD)^33,39^, being *γ*_*k*_ the singular values and ${{\bf{x}}}_{k}^{out}$ and ${{\bf{x}}}_{k}^{in}$ the related singular vectors (see SI, Sect. 2.4).

### Weighted networks

To extend our approach to weighted networks, one has to replace in Eqs (1–3) the adjacency matrix **A** with the matrix of the weights **W**, whose elements are defined as *w*_*ij*_ > 0 if there is a flux connecting *i* to *j*, zero otherwise. All the centrality measures in their weighted version are obtained as the solution of a matrix estimation exercise.

### Bipartite networks

Bipartite networks are characterised by two sets of nodes - **U** and **V** - with *E* edges connecting nodes between the two ensembles. These networks are described by the incidence matrix^2^ **B** whose elements *b*_*ij*_ define the relationship between the nodes *i* ∈ **U** and the nodes *j* ∈ **V**. In this case, the estimator ${\hat{B}}_{ij}$ will be a function of a property *x*_*i*_ of the nodes in the ensamble **U** and of a property *y*_*j*_ of the nodes in the ensamble **V** i.e., ${\hat{B}}_{ij}=f({x}_{i},{y}_{j})$. The centrality metrics obtained in Table 2 are straightforward extended to bipartite networks. By using the function *f* = *γx*_*i*_*y*_*j*_ and assuming a multiplicative error structure and an unbiased estimator, it is possible to recover the *Fitness*-*Complexity* algorithm, extensively used in characterising nations’ wellness^22,40^. Specifically, *x*_*i*_ represents the Fitness of the node *i* and *y*_*j*_ the Complexity of the node *j*.

## Results and Discussion

**Figure 1:**
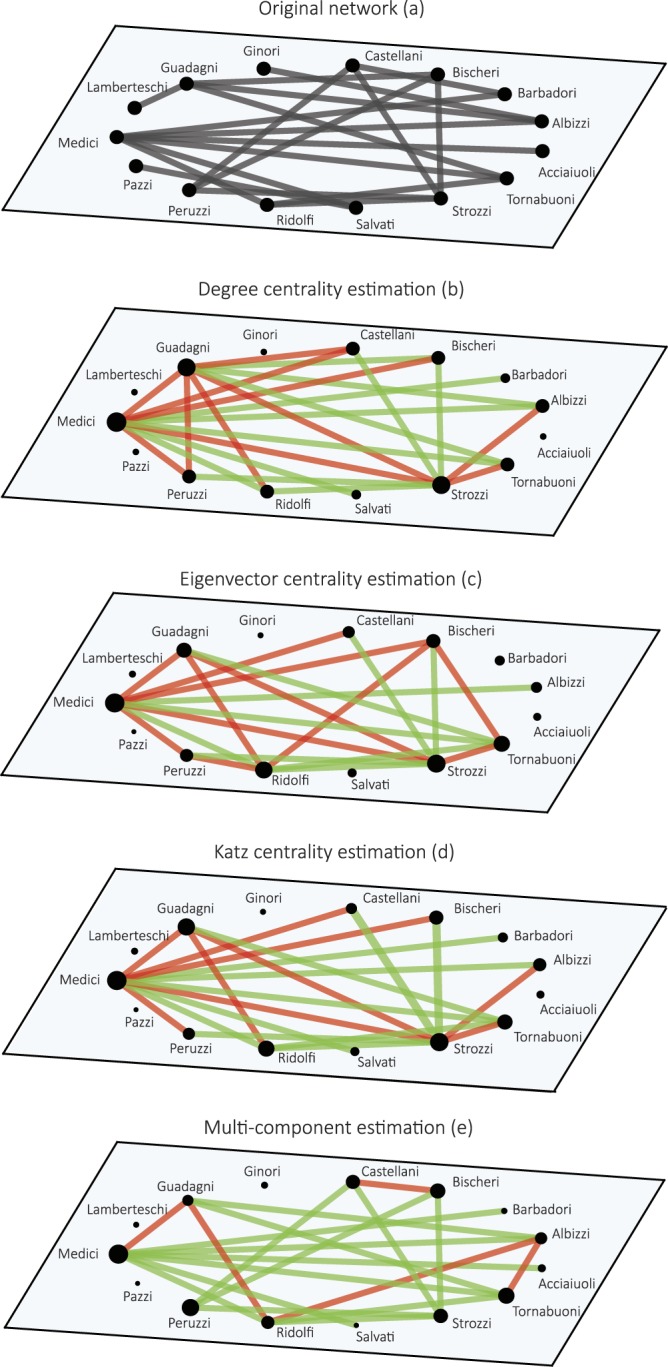
Estimation results for the undirected network of Florentine Intermarriage Relations, represented in panel (a). Panels (b–d) refer to the topology estimated by the degree, eigenvector, and Katz centrality, respectively. Panel (e) shows the estimated network as given by the multi-component estimator with two components (*s* = 2). In the figure, correctly estimated links are highlighted in green, while spurious links are red coloured. Nodes’ size in panels (b–e) is proportional to the position in the ranking resulting from the unique contribution, ordering the list from least to most central node. We plot in Fig. 1 only the *E* larger values of ${\hat{A}}_{ij}$, thus preserving in all the reconstructed networks the number *E* of edges of the real network. Exception is made when the *E*-th larger value of $\hat{{\bf{A}}}$ is a tie, in which case more than *E* edges are plotted. Rankings are available in the SI, Sect. 1.5.

We illustrate our new perspective starting in Fig. 1 with an analysis of the network of the Florentine Intermarriage Relations^41^. The network has 15 nodes representing the most notables Renaissance families in Florence connected by marriage relations (20 edges). Within our framework, the centrality measures have a counterpart in a link-estimation function, which allows to perform a visual and numerical comparison with the original network. We plot the original network in Fig. 1(a), and those resulting from the use of the one-component centrality measures in Fig. 1(b–d). The centrality-based estimations are performed using the functions reported in Table 1. For the computation of the Katz centrality, we used *α* = 0.5/*λ*_1_ following^42^, being *λ*_1_ the principal eigenvalue of **A** (see SI, Sect. 1.4). The network representation in Fig. 1(e) shows the result of the estimation provided by the multi-component estimator with *s* = 2. Figure 1 highlights the low agreement between the one-dimensional modelled networks and the real one. Several spurious and lacking links appear in the reconstructed graphs. The network representation is significantly improved when using the multi-component estimator (*s* = 2) in Fig. 1(e).

Besides the visual inspection, we compute the adjusted coefficient of determination ${R}_{a}^{2}$ between the original and the estimated matrices, **A** and $\hat{{\bf{A}}}$, in order to measure the quality of the estimation. ${R}_{a}^{2}$ is defined as

$${R}_{a}^{2}=1-(1-{R}^{2})\frac{{N}^{2}}{{N}^{2}-s\cdot N}=1-(1-{R}^{2})\frac{N}{N-s}.$$

The choice of ${R}_{a}^{2}$ as an error metric is consistent with the concept of unique contribution (see Eq. (4)). Moreover, this error measure is applicable to binary variables as well and the “adjusted” version of *R*^2^ allows one to compare the results obtained from distinct estimators and on differently sized networks. Notice that, while using ${R}_{a}^{2}$ instead of *R*^2^ is formally correct, the term *N*/(*N* − *s*) rapidly converges to 1 in large networks, making this correction negligible in some practical applications. For the Florentine Intermarriage Relations network, the adjusted determination coefficient for the multi-component estimator is ${R}_{a}^{2}=0.30$, while for the other estimators is around ${R}_{a}^{2}=0.07$, confirming the outcomes of the visual inspection.

**Figure 2:**
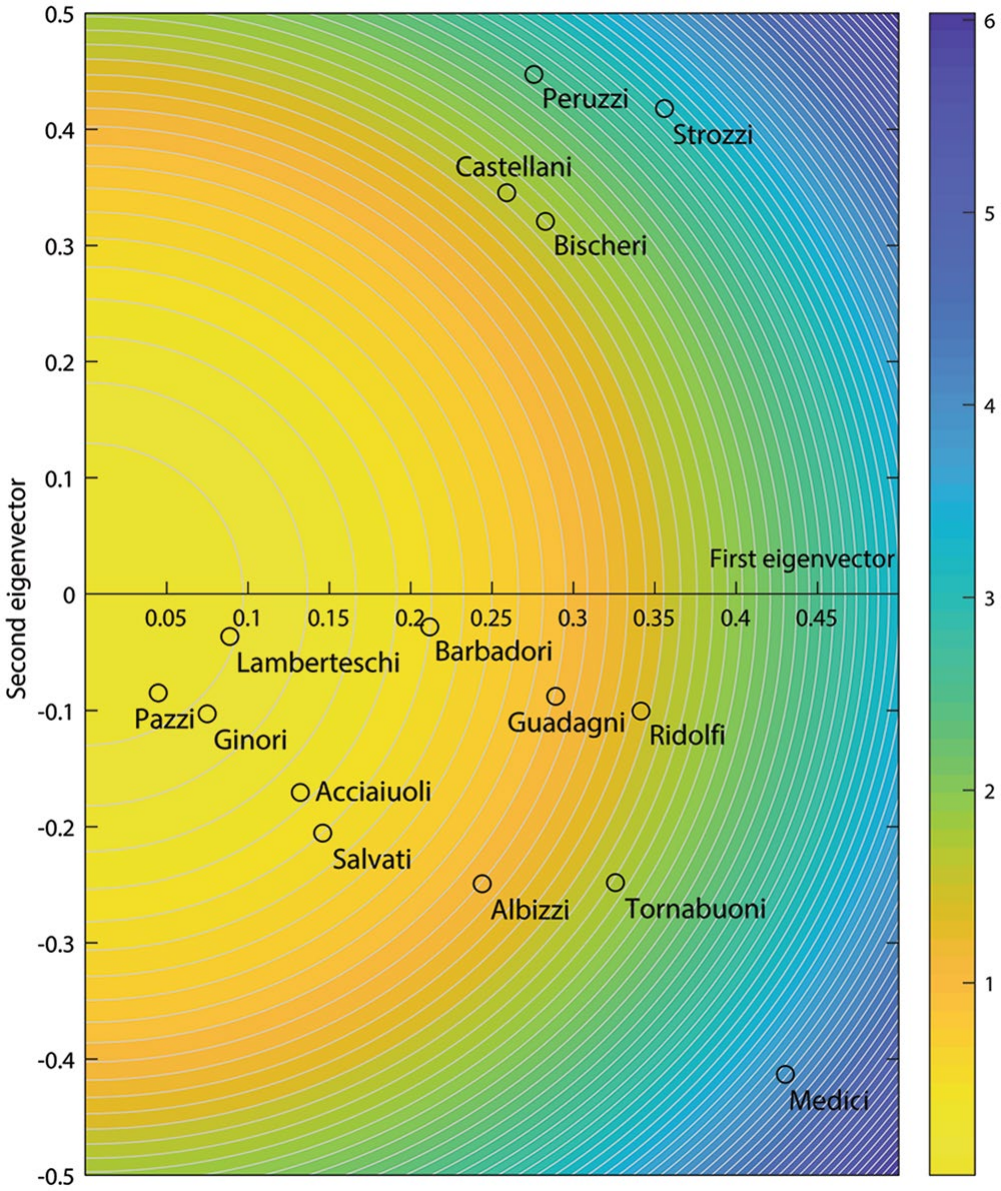
Contour plot of the unique contribution resulting from the application of Eq. (6) with *s* = 2. The contours range from lower values of unique contribution (in yellow) to larger values (in blue). The *x*_*i*,1_ values (corresponding to the components of the first eigenvector) are on the x-axis, while the values of *x*_*i*,2_ (related to the components of the eigenvector corresponding to the second eigenvalue, ordered following the method described in the SI, Sect. 1.5) are on the y-axis. The open circles correspond to the *x*_*i*,1_ and *x*_*i*,2_ values for the Florentine Intermarriage Relations network. Nodes with larger unique contribution are found further away from the origin.

The three classical centrality metrics (degree, eigenvector, Katz) produce different rankings of the Florentine families. While the *Medici* are always the top-ranked family, other families significantly change their position in the rankings (e.g., the ranking of the *Ridolfi* family changes from 3 to 7 when different methods are considered). By embracing our new perspective on network centrality it is possible to compare these rankings claiming that, despite the differences, from a statistical point of view the three metrics bring the same information about the topology of the network. The need to extend the centrality concept toward multiple dimensions manifestly emerges from Fig. 2. The second eigenvector distinctly identifies the group constituted by the families *Strozzi*-*Peruzzi*-*Castellani*-*Bischeri*, while highlighting how the *Medici* family is left alone by these four families. In this case the information brought by the second eigenvector is clearly relevant in determining the ranking of the nodes. In fact, the ranking in the case of Fig. 2 corresponds to the radial distance from the axes-origin. If one had considered only the first eigenvector, the *Ridolfi* family would have been ranked in the third position. The additional information carried by the second eigenvector, combined through the unique contribution, downgrades the *Ridolfi* family to the seventh position.

**Figure 3:**
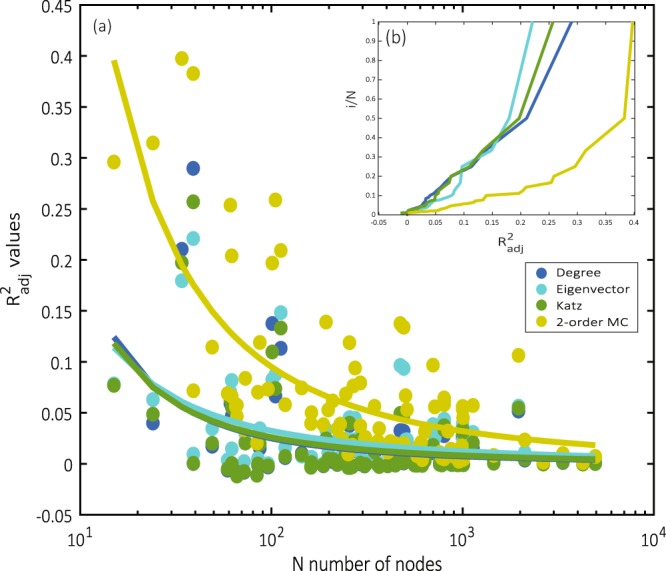
(**a**) Values of the coefficient of determination ${R}_{a}^{2}$, in semi-log scale obtained through the centrality-based estimators degree, eigenvector, Katz and multi-component (MC). Each dot refer to a network in the *Sparse Matrix* database^43^. Power-law curves are fitted to the data to facilitate visual comparison. (**b**) Cumulative frequency curves for the ${R}_{a}^{2}$ obtained by the four estimators.

The outcomes of the analysis of the network of the Florentine Intermarriage Relations are fully confirmed by a more extended analysis on 106 undirected networks, all freely available at https://sparse.tamu.edu/ ^43^. Our analysis includes all of the binary symmetric matrices available in the database sized *N* ≤ 1000. The list of the other networks included in our sample is given in the SI, Sect. 1.6. The values of ${R}_{a}^{2}$ obtained from the application of the functions in Table 1 are reported in Fig. 3. Two features clearly emerge. Firstly, the degree, the eigenvector and the Katz centrality systematically perform poorly when considered under the perspective of estimating the networks topology. This is essentially due to the compression of information from *N*^2^ to *N* implied by the matrix-estimation exercise, undermining the performance of the estimators. In general, ${R}_{a}^{2}$ decreases proportionally to the square root of *N*, following the behaviour of the standard deviation of the centrality-based estimators. Hence, the largest the size, the more information is lost during the estimation. The plot shows systematically higher values of ${R}_{a}^{2}$ resulting from the application of the two-components estimator Eq. (5). As expected, considering more node’s properties dramatically improves the estimation quality. Qualitatively similar results for directed networks are reported in the SI, Sect. 2.5.

A second key feature emerging from Fig. 3 is that the values of ${R}_{a}^{2}$ obtained from different one-component estimators are only slightly different from one another, and there is no evidence of one centrality measure outperforming the others. It follows that, despite the different nature of the metrics (i.e., the degree is a *local* measure of nodes’ importance, while the eigenvector and the Katz centrality are *global* measures^15^), all the metrics provide very similar and limited information about the topology of the networks. In this case, using different centrality metrics would not add new and divers information, resulting with redundancy of the metrics and therefore providing a further proof of their correlation^44^.

## Conclusions

This work introduced a different point of view about centrality, through which the evaluation of the importance of nodes is recast as a statistical-estimation problem. Here, centrality becomes the node-property through which one estimates the adjacency matrix of the network, breaking new ground in the way we understand node centrality. Many of the most commonly used centrality metrics can be deduced within this theoretical framework, thus paving the way for an unprecedented chance to quantitatively compare the performances of different centrality measures.

Aiming at showing the innovative power of our statistical perspective on centrality metrics, in this paper we focused on the application of this framework on monopartite networks and payed attention to the degree centrality and the eigenvector-based centrality measures. However, we stress that our approach is very general and should not be restricted to the examples reported above. In fact, this approach can be extended to other centrality measures, by changing the estimator function in Eq. (1), and/or the error structure – additive or multiplicative – and/or the matrix whereon the estimation procedure is carried out (either the adjacency matrix or a transformation of this one). Examples of this extension are the PageRank centrality^10^ and the Freeman closeness^6^. Within our framework, these two measures can be obtained through the application of the estimation procedure on the *Google matrix* **G**^10^ and on the *geodesic distance matrix* **D**^45^, respectively. Moreover, we argue that the estimator functions may also shed some light on the mathematical nature of the algorithms used to evaluate node centrality. In many cases, this would allow to find the exact analytic solution of the underlying mathematical maps, and thus avoiding tedious and imprecise iterative solutions.

Finally, the estimators could also explain the capability of the various algorithms to account for the nodes-nodes interactions. For example, by looking at the functions in Table 1, it is indeed clear that the degree centrality, obtained from a linear combination of the single properties of the nodes, cannot accommodate non-linear interactions among nodes. For this reason, the comparison of the performances of the various algorithms within our framework, could also be illuminating on the nature of the nodes interactions of a given system.

Tests on a large number of networks show that there are no outperforming one-dimensional, centrality-based estimators and that all the metrics provide poor information regarding networks’ topology. Our results, within the context of the still ongoing debate on the centrality metrics and the associated rankings (in several fields, see, e.g.^14,15,46–48^), provide further proofs that centrality metrics are highly correlated^42,44,49–52^ and that they provide similar information about the importance of the nodes. Within this new framework, a natural multi-component extension of node centrality emerges as a possible solution to improve the quality of the estimations and, subsequently, of node ranking. Our approach therefore provides a possible quantitative answer to the long-standing question “*what does it mean to be central in a network*?”.

## Electronic supplementary material

Supplementary Information

## Data Availability

The dataset used to perform this research is freely available on-line at *the SuiteSparse Matrix Collection*^43^ https://sparse.tamu.edu/. The authors are willing to provide further details upon request.
