# Supplementary material for: A change of perspective in network centrality
Source: Sci Rep. 2018 Oct 15;8:15269. doi: 10.1038/s41598-018-33336-8 (PMC6189051; doi:10.1038/s41598-018-33336-8)
Supplement: Supplementary file 1 — Supplementary Information [file 41598_2018_33336_MOESM1_ESM.pdf]

A change of perspective in network centrality - Supplementary Information

Carla Sciarra[1,*], Guido Chiarotti[1], Francesco Laio[1], and Luca Ridolfi[1]

[1]Department of Environmental, Land and Infrastructure Engineering, Politecnico di Torino, Corso Duca degli Abruzzi, 24, 10129 Torino, (IT)
[*]To whom correspondence and requests for materials should be addressed: carla.sciarra@polito.it

ABSTRACT

In this Supplementary Information, details about the mathematical results reported in the main text are provided. We start dealing with undirected networks, Sect. S1, and then consider directed ones, Sect. S2.

S1 Undirected networks

S1.1 General considerations

In this work, we recast the problem of evaluating the centrality of the nodes in a network as a matrix-estimation exercise. The estimator $\hat{A}_{ij}$ of the generic element A_{ij} of the adjacency matrix depends on the centrality x_i of the nodes, namely

$$\hat{A}_{ij} = f(x_i, x_j). \tag{S1.1}$$

For undirected networks, the adjacency matrix $\mathbf{A}$ is symmetric, i.e., $A_{ij} = A_{ji}$. In our framework, this entails that the arguments of any estimator function $\hat{A}_{ij}$ should be exchangeable, namely

$$\hat{A}_{ij} = f(x_i, x_j) = f(x_j, x_i).$$

The x_i values are found by minimising the sum of the squared (SE) residuals between the original element A_{ij} and its corresponding estimator $\hat{A}_{ij}$, with

$$SE = \sum_i \sum_j (A_{ij} - \hat{A}_{ij})^2 = \sum_i \sum_j (A_{ij} - f(x_i, x_j))^2. \tag{S1.2}$$

The minimisation procedure entails taking the derivative of SE with respect to the considered variable (say, x_k), and equaling it to zero. SE can be partitioned into two components: a first part which is independent of x_k (SE_0), and a second part depending on x_k (SE_k) i.e.,

$$SE = SE_0 + SE_k.$$

The derivative SE with respect to the variable x_k, being SE_0 independent of x_k, corresponds to the derivative of SE_k. Notice that SE_k only depends on the k-th row and column of the two matrices $\mathbf{A}$ and $\hat{\mathbf{A}}$, namely

$$SE_k = \sum_{i \neq k} \Big(A_{ik} - f(x_i, x_k)\Big)^2 + \sum_{j \neq k} \Big(A_{kj} - f(x_k, x_j)\Big)^2 + \Big(A_{kk} - f(x_k, x_k)\Big)^2, \tag{S1.3}$$

and the sums over the row and over the column coincide due to the symmetry of the matrix $\mathbf{A}$.

The derivative of the function SE with respect to the variable x_k, using Eq. (S1.3), is

$$\frac{\partial SE}{\partial x_k} = \frac{\partial SE_k}{\partial x_k} = 4 \sum_{i \neq k} \Big[A_{ik} - f(x_i, x_k)\Big] \frac{\partial f(x_i, x_k)}{\partial x_k} + 2 \Big[A_{kk} - f(x_k, x_k)\Big] \frac{\partial f(x_k, x_k)}{\partial x_k} = 0. \tag{S1.4}$$

Let us introduce the bound variable z_m which allows one to formalize more concisely the mathematics behind Eq. (S1.4). One has that

$$\frac{\partial f(x_i, x_k)}{\partial x_k} = \left. \frac{\partial f(x_i, z_m)}{\partial z_m} \right|_{z_m = x_k} \qquad \text{if} \qquad i \neq k \tag{S1.5}$$

and, if $i = k$,

$$\frac{\partial f(x_k,x_k)}{\partial x_k} = \left.\frac{\partial f(z_m,x_k)}{\partial z_m}\right|_{z_m=x_k} + \left.\frac{\partial f(x_k,z_m)}{\partial z_m}\right|_{z_m=x_k} = 2\left.\frac{\partial f(x_k,z_m)}{\partial z_m}\right|_{z_m=x_k}. \tag{S1.6}$$

Consider that the first equality in the second equation can be obtained by invoking the chain rule of derivation, with

$$\frac{\partial f(x,y(x))}{\partial x} = \frac{\partial f}{\partial x} + \frac{\partial f}{\partial y}\cdot\frac{\partial y}{\partial x}$$

setting $y = x$ afterwards, while the second equality holds because

$$\frac{\partial f(x_k,z_m)}{\partial z_m} = \frac{\partial f(z_m,x_k)}{\partial z_m}$$

due to the exchangeability of the arguments of the function f.

In this way, using Eq. (S1.6), Eq. (S1.4) becomes

$$\frac{\partial SS_k}{\partial x_k} = 4\sum_i [A_{ik} - f(x_i,x_k)]\cdot\left.\frac{\partial f(x_i,z_m)}{\partial z_m}\right|_{z_m=x_k}, \tag{S1.7}$$

where the index i runs over the range $[1,N]$. From Eq. (S1.7), x_k is obtained imposing the equivalence of the derivative to zero. An equation equivalent to Eq. (S1.7) is obtained for any centrality value x_i, $(i = 1,...,N)$.

Within the new perspective on network centrality described in this work, our definition of centrality is given through the analysis of the importance of the nodes in the estimation of A_{ij}, introducing the concept of unique contribution. We define the unique contribution of the generic node k as the difference between the coefficient of determination describing the goodness of fit of the estimation $\hat{A}_{ij}$ considering all the N centrality values, R^2_N, and the coefficient obtained by excluding the property of the node k, $R^2_{N\backslash k}$. This yields

$$UC_k = R^2_N - R^2_{N\backslash k} = \frac{SE_{N\backslash k} - SE_N}{TSS}, \tag{S1.8}$$

in which we have used the definition

$$R^2 = 1 - \frac{SE}{TSS},$$

where SE is defined in Eq. (S1.2). TSS is the variance of the adjacency matrix, i.e., $TSS = \sum_i\sum_j (A_{ij} - \bar{A})^2$, with $\bar{A}$ the mean of the matrix $\mathbf{A}$, namely

$$\bar{A} = \frac{\sum_i\sum_j A_{ij}}{N^2} = \frac{K_{tot}}{N^2}.$$

Hence

$$\begin{aligned} TSS &= \sum_i\sum_j (A_{ij} - \bar{A}_{ij})^2 \\ &= \sum_i\sum_j A^2_{ij} - 2\frac{K_{tot}}{N^2}\sum_i\sum_j A_{ij} + \frac{K^2_{tot}}{N^2} \end{aligned}$$

Since the elements of the adjacency matrix are either 1 or 0, $A^2_{ij} = A_{ij}$. This yields

$$TSS = K_{tot}\left(1 - \frac{K_{tot}}{N^2}\right). \tag{S1.9}$$

As obvious, TSS does not change with the exclusion of x_k. In order to evaluate the unique contribution, it is hence sufficient to compute the variation $\Delta SE = SE_{N\backslash k} - SE_N$ in Eq. (S1.8). For the sake of simplicity, we are not repeating the estimation procedure without considering the variable x_k, but we are merely setting $x_k = 0$ and keeping unchanged the other estimators x_i, $i \neq k$. Under these conditions, we can focus our attention on the k-th row and column only; ΔSE reads

$$\Delta SE = 2\sum_{i\neq k}\left[\Big(A_{ik} - f(x_i,0)\Big)^2 - \Big(A_{ik} - f(x_i,x_k)\Big)^2\right] + \Big(A_{kk} - f(0,0)\Big)^2 - \Big(A_{kk} - f(x_k,x_k)\Big)^2, \tag{S1.10}$$

that can be expressed as

$$\Delta SE = 2\sum_{i\neq k}\Big[f(x_i,0)^2 - f(x_i,x_k)^2 - 2f(x_i,0)A_{ik} + 2f(x_i,x_k)A_{ik}\Big] + f(0,0)^2 - f(x_k,x_k)^2 - 2f(0,0)A_{kk} + 2f(x_k,x_k)A_{kk}, \quad \text{(S1.11)}$$

or

$$\Delta SE = 2\sum_{i\neq k}\Big(f(x_i,0) - f(x_i,x_k)\Big)\Big(f(x_i,0) + f(x_i,x_k) - 2A_{ik}\Big) + \Big(f(0,0) - f(x_k,x_k)\Big)\Big(f(0,0) + f(x_k,x_k) - 2A_{kk}\Big). \quad \text{(S1.12)}$$

Within this paper, we consider networks with no self-loops, hence $A_{kk} = 0$.

The concept of centrality introduced with the unique contribution in Eq. (S1.8) may resemble the definition of the "induced" centrality measures[1] deriving from graph invariants. However, the two approaches are different. The induced centrality is obtained from the contribution that a single node provides into the computation of a given graph invariant. In this framework instead, any node is ranked according to its contribution to the estimation of the adjacency matrix of the graph.

S1.2 Degree centrality

Let us start by considering the estimator f_1 for undirected networks,

$$\hat{A}_{ij} = f_1(x_i,x_k) = a\left[x_i + x_k - \frac{1}{N}\right]. \quad \text{(S1.13)}$$

The derivative of the function f_1 with respect to x_k is

$$\left.\frac{\partial f_1(x_i,z_m)}{\partial z_m}\right|_{z_m=x_k} = a,$$

Applying Eq. (S1.7) one obtains

$$4a\sum_i\left[A_{ik} - a\left(x_i + x_k - \frac{1}{N}\right)\right] = 0.$$

Since $\sum_i A_{ik} = k_k$ is the degree of the node k, solving the equation for x_k yields $x_k = \frac{k_k}{aN}$. Assuming the vector of centralities to have unitary 1-norm i.e., $\sum_i x_i = 1$, one obtains

$$a = \frac{K_{tot}}{N}, \quad \text{(S1.14)}$$

finally yielding

$$x_k = \frac{k_k}{K_{tot}}. \quad \text{(S1.15)}$$

Eq. (S1.15) corresponds to rescaling the **degree centrality** by the total degree of the network.

S1.2.1 Unique contribution

From Eq. (S1.13), one has

$$f(x_i,0) = ax_i - \frac{a}{N},$$

and

$$f(0,0) = -\frac{a}{N}.$$

Using Eq. (S1.12), this provides

$$\begin{aligned}\Delta SE =& 2\sum_{i\neq k}(-ax_k)\left(2ax_i + ax_k - 2\frac{a}{N} - 2A_{ik}\right) + (-2ax_k)\left(2ax_k - 2\frac{a}{N}\right)\\ =& -2ax_k\sum_i\left(2ax_i + ax_k - 2\frac{a}{N} + 2A_{ik}\right) + 2a^2x_k^2.\end{aligned}$$

Some further algebra provides

$$\Delta SE = -2a^2 x_k^2 N + 4a x_k k_k + 2a^2 x_k^2.$$

Substituting the value of x_k as in Eq. (S1.15) and $a = K_{tot}/N$ in Eq. (S1.14), one obtains

$$\Delta SE = \frac{2(N+1)k_k^2}{N^2}$$

from which the unique contribution for the degree centrality is obtained,

$$UC_k = \frac{2(N+1)k_k^2}{N^2 TSS}. \tag{S1.16}$$

Since UC_k is a monotonic increasing function of k_k, ranking for increasing UC_k values provides the same ranking as the classical degree centrality.

S1.3 Eigenvector centrality

Consider the estimator for undirected network f_2 in Table 1, namely

$$\hat{A}_{ik} = f_2(x_i, x_k) = \gamma x_i x_k. \tag{S1.17}$$

The derivative of the function f_2 with respect to x_k is

$$\left.\frac{\partial f_2(x_i, z_m)}{\partial z_m}\right|_{z_m = x_k} = \gamma x_i,$$

Applying Eq. (S1.7) one obtains

$$4\sum_i \Big(A_{ik} - \gamma x_i x_k\Big)\gamma x_i = 0,$$

that solved for x_k provides

$$x_k = \frac{\sum_i A_{ik} x_i}{\gamma \sum_i x_i^2}.$$

We can assume the centrality vector to have unitary 2-norm (i.e., $\sum_i x_i^2 = 1$). This yields

$$x_k = \frac{1}{\gamma}\sum_i A_{ik} x_i. \tag{S1.18}$$

Eq. (S1.18) carries the same structure of the **eigenvector centrality**[2,3], where $\gamma = \lambda_1$ is the largest eigenvalue of $\mathbf{A}$. It is worth to notice that the relation in Eq. (S1.2), with the function Eq. (S1.17), recalls one of the relations from which Bonacich demonstrates the eigenvector centrality[4]. However, this is just a formal resemblance; in fact, Bonacich used the Principal Factor Method, assuming $\mathbf{A}$ to be a special correlation matrix and $\mathbf{x}$ to be its first principal factor associated to the largest eigenvalue (see[5,6] for details).

S1.3.1 Unique contribution

We use Eq. (S1.11), substituting f_2 for the generic function. In this case

$$f(x_i, 0) = f(0, 0) = 0,$$

from which Eq. (S1.11) becomes

$$\begin{aligned}\Delta SE &= 2\sum_{i \neq k}\Big[-\gamma^2 x_i^2 x_k^2 + 2\gamma x_i x_k A_{ik}\Big] - \gamma^2 x_k^4 + 2\gamma x_k^2 A_{kk} \\ &= 2\sum_i \Big[-\gamma^2 x_i^2 x_k^2 + 2\gamma x_i x_k A_{ik}\Big] + \gamma^2 x_k^4 - 2\gamma x_k^2 A_{kk}\end{aligned}$$

Since the 2-norm of the vector is unitary, and using (see Eq. (S1.18)),

$$\sum_i A_{ik} x_i = \gamma x_k,$$

one obtains

$$\Delta SE = 2\gamma^2 x_k^2 + \gamma^2 x_k^4,$$

in which the assumption $A_{kk} = 0$ is used. Therefore, the unique contribution of the node, according to the definition in Eq. (S1.8), is given by

$$UC_k = \frac{\gamma x_k^2}{TSS}\left(\gamma x_k^2 + 2\gamma\right). \tag{S1.19}$$

Since UC_k is a monotonic increasing function of x_k, ranking for increasing UC_k values provides the same ranking as the classical eigenvector centrality.

S1.4 Katz centrality

Consider the estimation function f_3 for undirected networks (see Table 1) assuming the parameter B to be negative,

$$\hat{A}_{ik} = f_3(x_i, x_k) = \gamma x_i x_k - B. \tag{S1.20}$$

The derivative of the function f_3 with respect to x_k, is

$$\left.\frac{\partial f_3(x_i, z_m)}{\partial x_k}\right|_{z_m = x_k} = \gamma x_i,$$

from which the derivative of the function SE according to Eq. (S1.7) is

$$4\sum_i \Big(A_{ik} - \gamma x_i x_k + B\Big)\gamma x_i = \tag{S1.21}$$

$$\sum_i A_{ik} x_i - \gamma x_k \sum_i x_i^2 + B\sum_i x_i = 0,$$

that, solved for x_k, provides

$$x_k = \frac{\sum_i A_{ik} x_i}{\gamma \sum_i x_i^2} + \frac{B\sum_i x_i}{\gamma \sum_i x_i^2}. \tag{S1.22}$$

We now introduce the *attenuation factor* α of the Katz centrality[7] and define the equivalences

$$\frac{1}{\gamma \sum_i x_i^2} = \alpha, \qquad \frac{B\sum_i x_i}{\gamma \sum_i x_i^2} = \beta \tag{S1.23}$$

obtaining

$$x_k = \alpha \sum_i A_{ik} x_i + \beta. \tag{S1.24}$$

Eq. (S1.24) corresponds to the definition of the Katz centrality measure[7], in which α is the attenuation factor whose value is $\alpha < 1/\lambda_1$, being λ_1 the largest eigenvalue of $\mathbf{A}$ and β is a constant, whose value is usually set to one[2]. Due to the constraint imposed by the form of the Katz centrality, the x_i values are always positive and greater than one; hence no assumptions can be made on the norms of the vector $\mathbf{x} = [x_1, \ldots, x_N]$.

S1.4.1 Unique contribution

Using the function f_3 in Eq. (S1.20), one has

$$f(x_i, 0) = f(0, 0) = -B.$$

Using the form of ΔSE as given in Eq. (S1.12) and substituting the values of the functions

$$f(x_i, 0) - f(x_i, x_k) = -\gamma x_i x_k, \qquad f(0, 0) - f(x_k, x_k) = -\gamma x_k^2,$$

one obtains

$$\Delta SE = 2\sum_{i\neq k}(-\gamma x_i x_k)(\gamma x_i x_k - 2B - 2A_{ik}) - \gamma x_k^2(\gamma x_k^2 - 2B - 2A_{kk})$$
$$= 2\sum_{i}(-\gamma^2 x_i^2 x_k^2 + 2\gamma B x_i x_k + 2\gamma x_i x_k A_{ik}) + \gamma^2 x_k^4 - 2\gamma B x_k^2,$$

where the assumption $A_{kk} = 0$ is used. Using the equivalences in Eq. (S1.23), and the one deriving from Eq. (S1.24),

$$\sum_i A_{ik} x_i = \frac{x_k}{\alpha} - \frac{\beta}{\alpha},$$

one obtains

$$\Delta SE = -2\gamma^2 x_k^2 \frac{1}{\alpha\gamma} + 4\gamma B x_k \frac{\beta}{\alpha B} + 4\gamma x_k\left(\frac{x_k}{\alpha} - \frac{\beta}{\alpha}\right) + \gamma^2 x_k^4 - 2B\gamma x_k^2$$
$$= 2\gamma\frac{x_k^2}{\alpha} + \gamma^2 x_k^4 - 2B\gamma x_k^2.$$

The unique contribution of the node, according to the definition Eq. (S1.8) is given by

$$UC_k = \frac{\gamma x_k^2}{TSS}\left(\gamma x_k^2 - 2B + \frac{2}{\alpha}\right). \quad \text{(S1.25)}$$

Since we have defined B to be negative, while γ and α are positive, UC_k is a monotonic increasing function of x_k and ranking for increasing UC_k values provides the same ranking as the classical Katz centrality.

S1.5 Multi-component centrality

Within our change of perspective, we introduced multi-component centrality metrics to improve the quality of the estimation. Within this framework, in case of undirected network, the multidimensional estimator reads

$$\hat{A}_{ij}(s) = \gamma_1 x_{i,1} x_{j,1} + \gamma_2 x_{i,2} x_{j,2} + \ldots + \gamma_s x_{i,s} x_{j,s} \quad \text{(S1.26)}$$
$$= \sum_{t=1}^{s} \gamma_t x_{i,t} x_{j,t}.$$

The estimator is a function of the s-dimensional vector embedding the s properties of the node that are considered for evaluating node's importance, namely $\hat{A}_{ij} = f(\mathbf{x}_i, \mathbf{x}_j)$, where $\mathbf{x}_i = [x_{i,1}, \ldots, x_{i,s}]$.

We assume the 2-norm of each vector $\mathbf{x}_t = [x_{1,t}, \ldots, x_{N,t}]$ to be unitary, i.e. $\sum_i x_{i,t}^2 = 1$. Moreover, we set an orthogonality condition between any two vectors $\mathbf{x}_t$ and $\mathbf{x}_{t^*}$, i.e.

$$\sum_i x_{i,t} \cdot x_{i,t^*} = 0, \qquad \forall t \neq t^*. \quad \text{(S1.27)}$$

The steps described for the one-component centrality can be adapted to the multidimensional setting. In this setting, we consider the contribution to SE of a generic variable x_{k,t^*}. As before, SE is partitioned into a part SE_0, which does not depend on x_{k,t^*}, and a part SE_{k,t^*}, which is a function of x_{k,t^*},

$$SE = SE_{0,t} + SE_{k,t^*}. \quad \text{(S1.28)}$$

The computation of the centrality values by minimisation of the $SE_{k,t*}$, entails computing Eq. (S1.7) accounting for each dimension considered i.e., $t = 1, \ldots, s$. The derivative of SE has the same form as Eq. (S1.7). Using the bound variable $\mathbf{z_m}$ one has

$$\left.\frac{\partial f(\mathbf{x}_i, \mathbf{z}_m)}{\partial z_{k,t^*}}\right|_{\mathbf{z}_m = \mathbf{x}_k} = \gamma_{t^*} x_{i,t^*}.$$

which gives

$$4\sum_i \left[A_{ik} - \sum_t \gamma_t x_{i,t} x_{k,t}\right]\gamma_{t^*} x_{i,t^*} = 0,$$

that is equivalent to

$$\sum_i A_{ik} x_{i,t^*} - \sum_t \gamma_t x_{k,t} \sum_i x_{i,t} \cdot x_{i,t^*} = 0.$$

Due to the orthonormality condition set in Eq. (S1.27), it holds

$$\begin{aligned}\sum_t \gamma_t x_{k,t} \sum_i x_{i,t} \cdot x_{i,t^*} &= \gamma_{t^*} x_{k,t^*} \sum_i x_{i,t^*} \cdot x_{i,t^*} \\ &= \gamma_{t^*} x_{k,t^*} \sum_i x_{i,t^*}^2 = \gamma_{t^*} x_{k,t^*}.\end{aligned}$$

Finally, for any component t, the centrality value reads

$$x_{k,t} = \frac{1}{\gamma_t} \sum_i A_{ik} x_{i,t}, \tag{S1.29}$$

which corresponds to computing the eigenvector $\mathbf{x}_t$ corresponding to the eigenvalue γ_t.

In Eq. (S1.26), the eigenvalues γ_t, and hence their corresponding eigenvectors $\mathbf{x}_t$, can be ordered according to their absolute value. This solution corresponds to the *Singular Value Decomposition* for symmetric matrices[8], being $\hat{\mathbf{A}}(s)$ the *s-order low-rank approximation* of the original adjacency matrix $\mathbf{A}$. The *Eckhart-Young-Mirsky theorem*[9] proofs that the total amount of explained variance VE of the *s-order low-rank approximation* equals the sum of the squares of the s eigenvalues, when the approximation is truncated at s, namely

$$VE(s) = \sum_{t=1}^{s} \gamma_t^2. \tag{S1.30}$$

For choosing the value of s, different strategies can be pursued (see, e.g.,[10] for a review of the criteria). For a given number of components s, at each component t^* added to the estimation, the total amount of explained variance increases by $\gamma_{t^*}^2$. Hence it holds

$$VE(t^*) - VE(t^* - 1) = \gamma_{t^*}^2 \tag{S1.31}$$

The total amount of unexplained variance VU is

$$\begin{aligned}VU(t^*) &= \sum_i \sum_j \left(A_{ij} - \hat{A}_{ij}(t^*)\right)^2 = TSS - VE(t^*) \\ &= TSS - \sum_{t=1}^{t^*} \gamma_{t^*}^2,\end{aligned} \tag{S1.32}$$

with TSS as in Eq. (S1.9).

The ordering of the eigenvalues, however, requires some additional considerations. In fact, Eq. (S1.30) ensures that the explained variance with s components is maximised by taking the first s eigenvalues, ordered in absolute values from the largest to the smallest. However, a consistency issue emerges when considering networks with no self loops. For these networks, the elements on the diagonal of $\mathbf{A}$ are zero. The estimated matrix has instead its diagonal elements different from zero, namely

$$\hat{A}_{ii}(s) = \sum_{t=1}^{s} \gamma_t x_{i,t}^2. \tag{S1.33}$$

This entails that, in order to provide a good description of the system, the eigenvalues should be ordered according to the total amount of explained variance they bring off-diagonal. In fact, Eq. (S1.30) can be partitioned in two terms, one pertaining with the diagonal D and the other with the off-diagonal OD terms, i.e.,

$$VE(s) = VE(s)_D + VE(s)_{OD}. \tag{S1.34}$$

We are therefore interested in ordering the eigenvalues so that the value $VE(t^*)_{OD}$ at each new added component t^* is maximised.

Consider the term $VE(t^*)_D$. Using Eq. (S1.32) and Eq. (S1.33), this term reads

$$VE(t^*)_D = TSS - \sum_i \Big(A_{ii} - \hat{A}_{ii}(t^*)\Big)^2 = TSS - \sum_i \Big(\hat{A}_{ii}(t^*)\Big)^2 \tag{S1.35}$$
$$= TSS - \sum_i \Big(\sum_{t=1}^{t^*} \gamma_t x_{i,t}^2\Big)^2$$
$$= TSS - \sum_i \Big(\sum_{t=1}^{t^*-1} \gamma_t x_{i,t}^2 + \gamma_{t^*} x_{i,t^*}^2\Big)^2$$
$$= TSS - \sum_i \Big(\sum_{t=1}^{t^*-1} \gamma_t x_{i,t}^2\Big)^2 - \gamma_{t^*}^2 \sum_i x_{i,t^*}^2 - \sum_i 2\gamma_{t^*} x_{i,t^*}^2 \Big(\sum_{t=1}^{t^*-1} \gamma_t x_{i,t}^2\Big).$$

Eq. (S1.35) entails that at each new component $t = t^*$ added to the estimation, the increment in the total amount of explained variance on the diagonal $\Delta VE(t^*)_D = VE(t^*)_D - VE(t^*-1)_D$ equals

$$\Delta VE(t^*)_D = -\gamma_{t^*}^2 \sum_i x_{i,t^*}^4 - 2\sum_i \gamma_{t^*} x_{i,t^*}^2 \sum_{t=1}^{t^*-1} \gamma_t x_{i,t}^2. \tag{S1.36}$$

Considering that the total amount of explained variance by the t^* component is $\gamma_{t^*}^2$ (see Eq. (S1.31)), one obtains from Eq. (S1.34) and Eq. (S1.36)

$$\Delta VE(t^*)_{OD} = \gamma_{t^*}^2 \Big(1 + \sum_i x_{i,t^*}^4\Big) + 2\sum_i \gamma_{t^*} x_{i,t^*} \sum_{t=1}^{t^*-1} \gamma_t x_{i,t}^2. \tag{S1.37}$$

Aiming at choosing the order in which the eigenvalues, and respective eigenvectors, should be embedded into the estimation Eq. (S1.26), one should maximise, at each step, the function in Eq. (S1.37). For $t = 1$ – i.e., for choosing the first eigenvalue and respective eigenvector – the function to be maximised is

$$\Delta VE(t^* = 1)_{OD} = \gamma_t^2 \Big(1 + \sum_i x_{i,t}^4\Big).$$

When $t = 2$, the second eigenvalue to be embedded into the function Eq. (S1.26) is the one that maximises the function

$$\Delta VE(t^* = 2)_{OD} = \gamma_{t=1}^2 \Big(1 + \sum_i x_{i,t=1}^4\Big) + 2\gamma_{t=1}\gamma_t \sum_i x_{i,t}^2 x_{i,t=1}^2.$$

In the main text, all of the results shown referring to the multi-component estimator and centrality have been processed according to the just described algorithm.

S1.5.1 Unique contribution

In the multi-component setting, the unique contribution is found accounting for all components $\mathbf{x}_t$, $t = (1, ..., s)$. In this case, excluding the generic node k from the estimation corresponds to nullifying all of its properties $x_{k,t}$, with $t = (1, ..., s)$. This yields

$$f(\mathbf{x}_i, 0) = f(0, 0) = 0.$$

Within this multi-component setting, Eq. (S1.12) becomes

$$\Delta SE = 2\sum_{i \neq k} \Big(-\sum_{t=1}^{s} \gamma_t x_{i,t} x_{k,t}\Big)\Big(\sum_{t=1}^{s} \gamma_t x_{i,t} x_{k,t} - 2A_{ik}\Big) - \Big(\sum_{t=1}^{s} \gamma_t x_{k,t}^2\Big)\Big(\sum_{t=1}^{s} \gamma_t x_{k,t}^2 - 2A_{kk}\Big)$$
$$= 2\sum_i \Big[-\Big(\sum_{t=1}^{s} \gamma_t x_{i,t} x_{k,t}\Big)^2 + 2A_{ik} \sum_{t=1}^{s} \gamma_t x_{i,t} x_{k,t}\Big] + \Big(\sum_{t=1}^{s} \gamma_t x_{k,t}^2\Big)^2.$$

that is equivalent to

$$\Delta SE = -2\sum_{t=1}^{s} \gamma_t^2 x_{k,t}^2 \sum_i x_{i,t}^2 + 4\sum_{t=1}^{s} \gamma_t x_{k,t} \sum_i A_{ik} x_{i,t} + \Big(\sum_{t=1}^{s} \gamma_t x_{k,t}^2\Big)^2$$

Using the orthonormality condition Eq. (S1.27) and Eq. (S1.29), the unique contribution in the case of the multi-component estimator is given by

$$UC(s)_k = 2\sum_{t=1}^{s} \gamma_t^2 x_{k,t}^2 + \Big(\sum_{t=1}^{s} \gamma_t x_{k,t}^2\Big)^2. \tag{S1.38}$$

Table S1. Rankings of the Florentine Renaissance Families resulting from the unique contribution of the centrality based estimation degree, eigenvector, Katz and multi-component.

Rankings of the Florentine Renaissance Families				
Families	Degree centrality	Eigenvector centrality	Katz centrality	Multi-component centrality ($s = 2$)
Acciaiuoli	13.5	12	12	12
Albizzi	6.5	9	7	8
Barbadori	10.5	10	10	10
Bischeri	6.5	6	6	5
Castellani	6.5	8	9	6
Ginori	13.5	14	14	13
Guadagni	2.5	5	3	9
Lamberteschi	13.5	13	13	14
Medici	1	1	1	1
Pazzi	13.5	15	15	15
Peruzzi	6.5	7	8	3
Ridolfi	6.5	3	4	7
Salvati	10.5	11	11	11
Strozzi	2.5	2	2	2
Tornabuoni	6.5	4	5	4

S1.6 Estimation results

Table S1 shows the rankings as given from the unique contribution of the centrality measures used to size the nodes in Figure 1 in the main text. In the table, nodes having the same centrality are treated as ties, i.e., they are assigned the same ranking. Nodes are ranked from the most central node, in position 1, to the least central.

We tested our framework on 106 networks freely available on the *Suite Sparse Matrix Collection*[11]. Our analysis includes all of the binary symmetric matrices collected in the database, sized $N \leq 1000$. Other networks included in the analysis are (as named as in the database):

- HB/dwt˙1005 - size $N = 1005$;
- HB/dwt˙1007 - size $N = 1007$;
- HB/jagmesh2 - size $N = 1009$;
- Arenas/email - size $N = 1133$;
- HB/bcspwr06 - size $N = 1454$;
- Rajat/rajat02 - size $N = 1960$;
- Barabasi/NotreDame˙yeast - size $N = 2114$;
- Gleich/minnesota - size $N = 2642$;
- HB/sstmodel - size $N = 3345$;
- AG-Monien/airfoil1 - size $N = 4253$;
- Newman/power - size $N = 4941$.

The results obtained from our tests are shown in Figure 3 in the main text.

S2 Directed Networks

S2.1 General considerations

Consider a directed network, whose adjacency matrix $\mathbf{A}$ is generally asymmetric. The estimator $\hat{A}_{ij}$ of the generic element A_{ij} now depends on both the *out* and *in* centrality of the nodes, namely

$$\hat{A}_{ij} = f(x_i^{out}, x_j^{in}). \tag{S2.1}$$

In the case of directed networks, the arguments of the function are exchangeable only on the diagonal, namely

$$f(x_k^{out}, x_k^{in}) = f(x_k^{in}, x_k^{out}).$$

The steps described for undirected networks to obtain the centrality values and to compute the unique contribution, Sect. S1, can be easily adapted to directed networks. Similarly to what described for undirected networks, the value SE can be partitioned into two components, a first part which is independent of $x_k^{out/in}$, SE_0, and a second part depending on $x_k^{out/in}$, SE_k, i.e.,

$$SE = SE_0 + SE_k.$$

When deriving SE with respect to any bound variable $z_m = x_k^{out/in}$, the contribution of the term SE_0 is null and the derivative of SE equals the derivative of SE_k. The term SE_k is

$$SE_k = \sum_{i \neq k} \Big(A_{ik} - f(x_i^{out}, x_k^{in})\Big)^2 + \sum_{j \neq k} \Big(A_{kj} - f(x_k^{out}, x_j^{in})\Big)^2 + \Big(A_{kk} - f(x_k^{out}, x_k^{in})\Big)^2. \tag{S2.2}$$

Notice that this term only depends on the k-th row and column of the two matrices $\mathbf{A}$ and $\hat{\mathbf{A}}$. Separating the row and column contribution, the derivatives of SE_k with respect to the variables x_k^{out} and x_k^{in}, using the bound variable z_m, are

$$\frac{\partial SE_k}{\partial x_k^{out}} = 2 \sum_{j \neq k} \Big[A_{kj} - f(x_k^{out}, x_j^{in})\Big] \cdot \frac{\partial f(z_m, x_j^{in})}{\partial z_m}\bigg|_{z_m = x_k^{out}} + 2\Big[A_{kk} - f(x_k^{out}, x_k^{in})\Big] \cdot \frac{\partial f(z_m, x_k^{in})}{\partial z_m}\bigg|_{z_m = x_k^{out}} = 0, \tag{S2.3}$$

and

$$\frac{\partial SE_k}{\partial x_k^{in}} = 2 \sum_{i \neq k} \Big[A_{ik} - f(x_i^{out}, x_k^{in})\Big] \cdot \frac{\partial f(x_i^{out}, z_m)}{\partial z_m}\bigg|_{z_m = x_k^{in}} + 2\Big[A_{kk} - f(x_k^{out}, z_m)\Big] \cdot \frac{\partial f(x_i^{out}, z_m)}{\partial z_m}\bigg|_{z_m = x_k^{in}} = 0. \tag{S2.4}$$

In Eq. (S2.3) and Eq. (S2.4), both the terms $i = k$ and $j = k$ can be included into the sums. Hence

$$\frac{\partial SE_k}{\partial x_k^{out}} = 2 \sum_{j} \Big[A_{kj} - f(x_k^{out}, x_j^{in})\Big] \cdot \frac{\partial f(z_m, x_j^{in})}{\partial z_m}\bigg|_{z_m = x_k^{out}} = 0, \tag{S2.5}$$

and

$$\frac{\partial SE_k}{\partial x_k^{in}} = 2 \sum_{i} \Big[A_{ik} - f(x_i^{out}, x_k^{in})\Big] \cdot \frac{\partial f(x_i^{out}, z_m)}{\partial z_m}\bigg|_{z_m = x_k^{in}} = 0. \tag{S2.6}$$

The unique contribution is found through Eq. (S1.8), hence computing $\Delta SE = SE_{N \setminus k} - SE_N$. In directed networks, nodes are characterised by two properties. Within this framework, the unique contribution can be computed with respect to one of the properties, or at the need, with respect to both ones. In the first case, one finds the *in*-centrality (or the *out*-centrality) of the node. In the second case the overall centrality of the node is obtained.

If both properties are considered in the computation, we can define ΔSE as

$$\Delta SE^{tot} = \sum_{i \neq k} \left[\Big(A_{ik} - f(x_i^{out}, 0)\Big)^2 - \Big(A_{ik} - f(x_i^{out}, x_k^{in})\Big)^2\right] + \sum_{j \neq k} \left[\Big(A_{kj} - f(0, x_j^{in})\Big)^2 - \Big(A_{ik} - f(x_k^{out}, x_j^{in})\Big)^2\right] \\ + \Big(A_{kk} - f(0,0)\Big)^2 - \Big(A_{kk} - f(x_k^{out}, x_k^{in})\Big)^2, \tag{S2.7}$$

in which we consider the exclusion of the properties x_k^{out} and x_k^{in} to be equivalent to setting $x_k^{out} = x_k^{in} = 0$. Eq. (S2.7) can be expressed as

$$
\begin{aligned}
\Delta SE^{tot} =& \sum_{i \neq k} \Big[f(x_i^{out},0)^2 - f(x_i^{out},x_k^{in})^2 - 2f(x_i^{out},0)A_{ik} + 2f(x_i^{out},x_k^{in})A_{ik} \Big] + \\
& \sum_{j \neq k} \Big[f(0,x_j^{in})^2 - f(x_k^{out},x_j^{in})^2 - 2f(0,x_j^{in})A_{kj} + 2f(x_k^{out},x_j^{in})A_{kj} \Big] \\
& + f(0,0)^2 - f(x_k^{out},x_k^{in})^2 - 2f(0,0)A_{kk} + 2f(x_k^{out},x_k^{in})A_{kk},
\end{aligned} \tag{S2.8}
$$

or

$$
\begin{aligned}
\Delta SE^{tot} =& \sum_{i \neq k} \Big(f(x_i^{out},0) - f(x_i^{out},x_k^{in}) \Big) \Big(f(x_i^{out},0) + f(x_i^{out},x_k^{in}) - 2A_{ik} \Big) \\
& + \sum_{j \neq k} \Big(f(0,x_j^{in}) - f(x_k^{out},x_j^{in}) \Big) \Big(f(0,x_j^{in}) + f(x_k^{out},x_j^{in}) - 2A_{kj} \Big) \\
& + \Big(f(0,0) - f(x_k^{out},x_k^{in}) \Big) \cdot \Big(f(0,0) - f(x_k^{out},x_k^{in}) - 2A_{kk} \Big)
\end{aligned} \tag{S2.9}
$$

The unique contribution is then found deploying the expression in Eq. (S2.8) or Eq. (S2.9), and applying the definition in Eq. (S1.8).

To compute the unique contribution with respect to one of the two properties entails considering, in Eq. (S2.8) or Eq. (S2.9), only the terms on the dimension related to the specific property at hand. Hence, the k-th row (sum over j) for the *out* centrality of the node k and the k-th column (sum over i) for its *in* centrality. In formulas

$$
\begin{aligned}
\Delta SE^{out} =& \sum_{j} \Big[f(0,x_j^{in})^2 - f(x_k^{out},x_j^{in})^2 - 2f(0,x_j^{in})A_{kj} + 2f(x_k^{out},x_j^{in})A_{kj} \Big] \\
=& \sum_{j} \Big(f(0,x_j^{in}) - f(x_k^{out},x_j^{in}) \Big) \Big(f(0,x_j^{in}) + f(x_k^{out},x_j^{in}) - 2A_{kj} \Big),
\end{aligned} \tag{S2.10}
$$

and

$$
\begin{aligned}
\Delta SE^{in} =& \sum_{i} \Big[f(x_i^{out},0)^2 - f(x_i^{out},x_k^{in})^2 - 2f(x_i^{out},0)A_{ik} + 2f(x_i^{out},x_k^{in})A_{ik} \Big] \\
=& \sum_{i} \Big(f(x_i^{out},0) - f(x_i^{out},x_k^{in}) \Big) \Big(f(x_i^{out},0) + f(x_i^{out},x_k^{in}) - 2A_{ik} \Big),
\end{aligned} \tag{S2.11}
$$

In the following, we consider networks with no self-loops, hence $A_{kk} = 0$.

S2.2 Degree centrality

Consider the function f_1

$$
\hat{A}_{ij} = f_1(x_i^{out}, x_k^{in}) = a \Big[x_i^{out} + x_k^{in} - \frac{1}{N} \Big]. \tag{S2.12}
$$

The derivatives of the function f_1 with respect to both properties x_k^{out} and x_k^{in} are

$$
\frac{\partial f_1}{\partial x_k^{out}} = \frac{\partial f_1}{\partial x_k^{in}} = a.
$$

Applying Eq. (S2.5) and Eq. (S2.6) one obtains

$$
2a \sum_{i} \Big[A_{ik} - a \Big(x_i^{out} + x_k^{in} - \frac{1}{N} \Big) \Big] = 0,
$$

and

$$
2a \sum_{j} \Big[A_{kj} - a \Big(x_k^{out} + x_j^{in} - \frac{1}{N} \Big) \Big] = 0,
$$

in which $\sum_i A_{ik} = k_k^{in}$ is the in-degree of the node k and $\sum_j A_{kj} = k_k^{out}$ is its out-degree. Solving both equations for the properties x_k^{out} and x_k^{in} yields

$$x_k^{in} = \frac{k_k^{in}}{aN}$$

and

$$x_k^{out} = \frac{k_k^{out}}{aN}.$$

Assuming the vectors of centralities $\mathbf{x}^{out}$ and $\mathbf{x}^{in}$ to have unitary 1-norm, i.e., $\sum_i x_i^{out} = \sum_i x_i^{in} = 1$, one obtains $a = K_{tot}/N$ as in Eq. (S1.14), finally yielding

$$x_k^{in} = \frac{k_k^{in}}{K_{tot}}, \tag{S2.13a}$$

$$x_k^{out} = \frac{k_k^{out}}{K_{tot}}. \tag{S2.13b}$$

Eq. (S2.13b)-Eq. (S2.13a) correspond to rescaling the **out-degree** and **in-degree** by the total degree of the network.

S2.2.1 Unique contribution

Let us start from the computation of the total unique contribution i.e., the UC of the node k when its properties *out* and *in* are considered together. From Eq. (S2.12), one has

$$\begin{aligned} f(x_i^{out}, 0) &= a x_i^{out} - \frac{a}{N}; \\ f(0, x_j^{in}) &= a x_j^{in} - \frac{a}{N}; \\ f(0,0) &= -\frac{a}{N}. \end{aligned}$$

Using Eq. (S2.9), one obtains

$$\begin{aligned} \Delta SE^{tot} =& \sum_{i \neq k} (-a x_k^{in}) \left(2a x_i^{out} + a x_k^{in} - 2\frac{a}{N} - 2A_{ik} \right) + \sum_{j \neq k} (-a x_k^{out}) \left(2a x_j^{in} + a x_k^{out} - 2\frac{a}{N} - 2A_{kj} \right) \\ &+ (-a x_k^{out} - a x_k^{in}) \left(a x_k^{out} + a x_k^{in} - 2\frac{a}{N} - 2A_{kk} \right) \\ =& - a x_k^{in} \sum_i \left(-2a x_i^{out} - a x_k^{in} + 2\frac{a}{N} + 2A_{ik} \right) - a x_k^{out} \sum_j \left(-2a x_j^{in} - a x_k^{out} + 2\frac{a}{N} + 2A_{kj} \right) + 2a^2 x_k^{out} x_k^{in}, \end{aligned}$$

in which the assumption $A_{kk} = 0$ is used. Substituting the values of x_k^{out} and x_k^{in} according to Eq. (S2.13), and considering $a = K_{tot}/N$, some algebra gives

$$\Delta SE^{tot} = \frac{(k_k^{in})^2 + (k_k^{out})^2}{N} + \frac{2k_k^{in} k_k^{out}}{N^2}$$

from which the unique contribution is obtained

$$UC_k^{tot} = \frac{1}{TSS} \left[\frac{(k_k^{in})^2 + (k_k^{out})^2}{N} + \frac{2k_k^{in} k_k^{out}}{N^2} \right] \tag{S2.14}$$

The unique contribution obtained by separately considering the property *out* or *in* is found applying Eq. (S2.10) - Eq. (S2.11), respectively. In this case one obtains

$$UC_k^{out} = \frac{(k_k^{out})^2}{NTSS}, \tag{S2.15}$$

$$UC_k^{in} = \frac{(k_k^{in})^2}{NTSS}. \tag{S2.16}$$

Both the formulations in Eq. (S2.15) and Eq. (S2.16) are monotonic increasing function of x_k^{out} and of x_k^{in}, respectively. Hence, ranking for increasing UC_k^{out} and UC_k^{in} values provide the same ranking as the classical in and out degree centrality.

S2.3 Hub-authority centrality

Consider the estimator for directed network f_2 in Table 2, namely

$$\hat{A}_{ik} = f_2(x_i^{out}, x_k^{in}) = \gamma x_i^{out} x_k^{in}. \tag{S2.17}$$

Clearly,

$$\frac{\partial f_2}{\partial x_k^{out}} = \gamma x_j^{out}, \qquad \frac{\partial f_2}{\partial x_k^{in}} = \gamma x_i^{in}.$$

Applying Eq. (S2.5) and Eq. (S2.6) one obtains

$$\begin{cases} \frac{\partial SE}{\partial x_k^{out}} = 2\sum_j (\gamma A_{kj} x_j^{in} - \gamma^2 x_k^{out} (x_j^{in})^2) = 0, \\ \frac{\partial SE}{\partial x_k^{in}} = 2\sum_i (\gamma A_{ik} x_i^{out} - \gamma^2 (x_i^{out})^2 x_k^{in}) = 0. \end{cases}$$

that, solved with respect to the properties x_k^{out} and x_k^{in}, within the assumption of unitary 2-norm of the vectors, i.e. $\sum_i (x_i^{out})^2 = 1$ and $\sum_j (x_j^{in})^2 = 1$, yields

$$\begin{cases} x_k^{out} = \frac{1}{\gamma} \sum_j A_{kj} x_j^{in}, \\ x_k^{in} = \frac{1}{\gamma} \sum_i A_{ik} x_i^{out}. \end{cases} \tag{S2.18}$$

In matrix form,

$$\begin{cases} \gamma \mathbf{x}^{out} = \mathbf{A} \mathbf{x}^{in}, \\ \gamma \mathbf{x}^{in} = \mathbf{A}^{\mathbf{T}} \mathbf{x}^{out}. \end{cases}$$

Some algebra gives

$$\begin{cases} \gamma^2 \mathbf{x}^{out} = \mathbf{A}\mathbf{A}^{\mathbf{T}} \mathbf{x}^{out}, \\ \gamma^2 \mathbf{x}^{in} = \mathbf{A}^{\mathbf{T}} \mathbf{A} \mathbf{x}^{in}. \end{cases}$$

Introducing the matrices $\mathbf{C} = \mathbf{A}^{\mathbf{T}}\mathbf{A}$ and $\mathbf{D} = \mathbf{A}\mathbf{A}^{\mathbf{T}}$, one has

$$\gamma^2 \mathbf{x}^{out} = \mathbf{D} \mathbf{x}^{out}, \tag{S2.19a}$$

$$\gamma^2 \mathbf{x}^{in} = \mathbf{C} \mathbf{x}^{in}. \tag{S2.19b}$$

Eq. (S2.19) states that $\mathbf{x}^{out}$ and $\mathbf{x}^{in}$ are the dominant eigenvectors of the matrices $\mathbf{D}$ and $\mathbf{C}$, respectively, associated to the principal eigenvalue of the two matrices, such that $\gamma^2 = \lambda_1(\mathbf{C}) = \lambda_1(\mathbf{D}) = \sigma_1^2(\mathbf{A})$[8,12], being σ_1 the principal singular value of the matrix $\mathbf{A}$. The formulation in Eq. (S2.19) matches the **HITS algorithm**[13], used to identify *hubs* and *authorities* in networks.

S2.3.1 Unique contribution

First, consider the unique contribution to be computed with respect to both the properties. Using Eq. (S2.17), one has

$$f(x_i^{out}, 0) = f(0, x_j^{in}) = f(0,0) = 0,$$

from which Eq. (S2.8) becomes

$$\begin{aligned} \Delta SE^{tot} &= \sum_{i \neq k} \left[-(\gamma x_i^{out} x_k^{in})^2 + 2\gamma x_i^{out} x_k^{in} A_{ik} \right] + \sum_{j \neq k} \left[-(\gamma x_k^{out} x_j^{in})^2 + 2\gamma x_k^{out} x_j^{in} A_{kj} \right] + \left[-(\gamma x_k^{out} x_k^{in})^2 + 2\gamma x_k^{out} x_k^{in} A_{kk} \right] \\ &= \sum_i \left[-(\gamma x_i^{out} x_k^{in})^2 + 2\gamma x_i^{out} x_k^{in} A_{ik} \right] + \sum_j \left[-(\gamma x_k^{out} x_j^{in})^2 + 2\gamma x_k^{out} x_j^{in} A_{kj} \right] - \left[-(\gamma x_k^{out} x_k^{in})^2 \right], \end{aligned}$$

in which the assumption $A_{kk}=0$ is used. Some algebra provides

$$\Delta SE^{tot} = -\gamma(x_k^{in})^2\sum_i(x_i^{out})^2 + 2\gamma x_k^{in}\sum_i x_i^{out}A_{ik} - \gamma(x_k^{out})^2\sum_j(x_j^{in})^2 + 2\gamma x_k^{out}\sum_j A_{kj}x_j^{in} + (\gamma x_k^{out}x_k^{in})^2. \quad (S2.20)$$

Since the 2-norm of the vectors $\mathbf{x}^{out}$ and $\mathbf{x}^{in}$ is unitary and using Eq. (S2.18), one has

$$\Delta SE^{tot} = \gamma^2(x_k^{out})^2 + \gamma^2(x_k^{in})^2 + (\gamma x_k^{out}x_k^{in})^2.$$

The total unique contribution of the node k applying the definition Eq. (S1.8) is

$$UC_k^{tot} = \frac{\gamma^2(x_k^{out})^2 + \gamma^2(x_k^{in})^2 + (\gamma x_k^{out}x_k^{in})^2}{TSS}. \quad (S2.21)$$

In order to compute the unique contribution accounting separately for the properties *out* or *in*, Eq. (S2.10) - Eq. (S2.11) are used

$$\Delta SE^{out} = \sum_j\left[-(\gamma x_k^{out}x_j^{in})^2 + 2\gamma x_k^{out}x_j^{in}A_{kj}\right],$$

and

$$\Delta SE^{in} = \sum_i\left[-(\gamma x_i^{out}x_k^{in})^2 + 2\gamma x_i^{out}x_k^{in}A_{ik}\right].$$

Going through the same algebra as for Eq. (S2.20) and applying the definition of unique contribution, one obtains

$$UC_k^{out} = \frac{\gamma^2(x_k^{out})^2}{TSS}. \quad (S2.22)$$

and

$$UC_k^{in} = \frac{\gamma^2(x_k^{in})^2}{TSS}. \quad (S2.23)$$

Both the formulations in Eq. (S2.22) and Eq. (S2.23) are monotonic increasing function of x_k^{out} and of x_k^{in}, respectively. Hence, ranking for increasing UC_k^{out} and UC_k^{in} values provide the same ranking as the classical hub-authority algorithm.

S2.4 Multi-component centrality

In the case of directed networks, the multi-component estimator is a function of the s-dimensional vectors $\mathbf{x}_i^{out}$ and $\mathbf{x}_j^{in}$ considered for evaluating node's importance, namely $\hat{A}_{ij} = f(\mathbf{x}_i^{out}, \mathbf{x}_j^{in})$, where $\mathbf{x}_i^{out} = [x_{i,1}^{out}, ..., x_{i,s}^{out}]$ and $\mathbf{x}_j^{in} = [x_{j,1}^{in}, ..., x_{i,s}^{in}]$. Within this framework, the multidimensional estimator is

$$\begin{aligned}\hat{A}_{ij}(s) &= \gamma_1 x_{i,1}^{out}x_{j,1}^{in} + \gamma_2 x_{i,2}^{out}x_{j,2}^{in} + ... + \gamma_s x_{i,s}^{out}x_{j,s}^{in} \\ &= \sum_{t=1}^{s}\gamma_s x_{i,t}^{out}x_{j,t}^{in}.\end{aligned} \quad (S2.24)$$

We assume the 2-norm of each vector $\mathbf{x}_t^{out} = [x_{1,t}^{out}, ..., x_{N,t}^{out}]$ and $\mathbf{x}_{i,t}^{in} = [x_{1,t}^{in}, ..., x_{N,t}^{in}]$ is unitary i.e., $\sum_i(x_{i,t}^{out})^2 = \sum_i(x_{i,t}^{in})^2 = 1$. Moreover, we set an orthogonality condition between any two vectors $\mathbf{x}_t^{out/in}$ and $\mathbf{x}_{t^*}^{out/in}$, i.e.

$$\sum_i x_{i,t}^{out}\cdot x_{i,t^*}^{out} = 0, \qquad \forall t \neq t^*, \quad (S2.25)$$

$$\sum_i x_{i,t}^{in}\cdot x_{i,t^*}^{in} = 0, \qquad \forall t \neq t^*. \quad (S2.26)$$

Similarly to Sect. S1, in this multi-component setting the function SE is expressed as Eq. (S1.28). In order to compute the centrality values, it is necessary to derive the function $SE_{k,t}$ accounting for the s dimensions embedded in the estimators. The derivatives of the multi-component estimator Eq. (S2.24) with respect to the variables $\mathbf{x}_{k,t^*}^{out}$ and $\mathbf{x}_{k,t^*}^{in}$ at any order t^* are

$$\left.\frac{\partial f(\mathbf{x}_i^{out}, \mathbf{z}_m)}{\partial z_{k,t^*}}\right|_{\mathbf{z}_m=\mathbf{x}_k^{in}} = \gamma_{t^*}x_{i,t^*}^{out},$$

and

$$\left.\frac{\partial f(\mathbf{z}_m, \mathbf{x}_j^{in})}{\partial z_{k,t^*}^{in}}\right|_{\mathbf{z}_m=\mathbf{x}_k^{out}} = \gamma_{t^*} x_{j,t^*}^{in},$$

that, introduced in Eq. (S2.5) and Eq. (S2.6), provide

$$2\sum_i \left[A_{ik} - \sum_t \gamma_t x_{i,t}^{out} x_{k,t}^{in}\right] \gamma_{t^*} x_{i,t^*}^{out} =$$
$$\sum_i A_{ik} x_{i,t^*}^{out} - \sum_t \gamma_t x_{k,t}^{in} \sum_i x_{i,t^*}^{out} x_{i,t}^{out} = 0$$

and

$$2\sum_j \left[A_{kj} - \sum_t \gamma_t x_{k,t}^{out} x_{j,t}^{in}\right] \gamma_{t^*} x_{j,t^*}^{in} =$$
$$\sum_j A_{kj} x_{j,t^*}^{in} - \sum_t \gamma_t x_{k,t}^{out} \sum_j x_{j,t^*}^{in} x_{j,t}^{in} = 0$$

Using the conditions of othonormality, Eq. (S2.25) - Eq. (S2.26), some algebra provides

$$\begin{cases} x_{k,t}^{out} = \frac{1}{\gamma_t} \sum_j A_{kj} x_{j,t}^{in}, \\ x_{k,t}^{in} = \frac{1}{\gamma_t} \sum_i A_{ik} x_{i,t}^{out}. \end{cases} \tag{S2.27}$$

Eq. (S2.27) states that at any order t, the vectors $\mathbf{x}_t^{out} = [x_{1,t}^{out}, \ldots, x_{N,t}^{out}]$ and $\mathbf{x}_t^{in} = [x_{1,t}^{in}, \ldots, x_{N,t}^{in}]$ are the left and right singular vectors associated to the singular value γ_t, respectively.

The estimation provided in Eq. (S2.24) is the *s-order low-rank approximation* of the original adjacency matrix $\hat{A}$.

S2.4.1 Unique contribution

In the multi-component setting for directed networks, the unique contribution is found accounting for the s dimensions embedded in the estimator function f (see Eq. (S2.24)). In this case, when excluding the generic node k from the estimation, all the properties $x_{k,t}^{out}$ and $x_{k,t}^{in}$, with $t = (1, \ldots, s)$, are nullified. This yields

$$f(\mathbf{x}_i^{out}, 0) = f(0, \mathbf{x}_j^{in}) = f(0,0) = 0.$$

Within this multi-component setting, the unique contribution can be computed with respect to both the properties $\mathbf{x}_{k,t}^{out}$ and $\mathbf{x}_{k,t}^{in}$, or with respect to one of the two.

If both the properties are considered, Eq. (S2.9) holds, providing

$$\Delta SE^{tot} = \sum_{i\neq k}\left(-\sum_{t=1}^{s} \gamma_t x_{i,t}^{out} x_{k,t}^{in}\right)\left(\sum_{t=1}^{s} \gamma_t x_{i,t}^{out} x_{k,t}^{in} - 2A_{ik}\right) + \sum_{j\neq k}\left(-\sum_{t=1}^{s} \gamma_t x_{k,t}^{out} x_{j,t}^{in}\right)\left(\sum_{t=1}^{s} \gamma_t x_{k,t}^{out} x_{j,t}^{in} - 2A_{kj}\right)$$
$$+ \left(-\sum_{t=1}^{s} \gamma_t x_{k,t}^{out} x_{k,t}^{in}\right)\left(\sum_{t=1}^{s} \gamma_t x_{k,t}^{out} x_{k,t}^{in} - 2A_{kk}\right).$$

that is equivalent to

$$\Delta SE^{tot} = \sum_i \left[-\left(\sum_{t=1}^{s} \gamma_t x_{i,t}^{out} x_{k,t}^{in}\right)^2 + 2A_{ik} \sum_{t=1}^{s} \gamma_t x_{i,t}^{out} x_{k,t}^{in}\right] + \sum_j \left[-\left(\sum_{t=1}^{s} \gamma_t x_{k,t}^{out} x_{j,t}^{in}\right)^2 - 2A_{kj} \sum_{t=1}^{s} \gamma_t x_{k,t}^{out} x_{j,t}^{in}\right] + \left(\sum_{t=1}^{s} \gamma_t x_{k,t}^{out} x_{k,t}^{in}\right)^2.$$

Some algebra provides

$$\Delta SE^{tot} = -\sum_{t=1}^{s} \gamma_t (x_{k,t}^{in})^2 \sum_i (x_{i,t}^{out})^2 + 2\sum_{t=1}^{s} \gamma_t x_{k,t}^{in} \sum_i A_{ik} x_{i,t}^{out} - \sum_{t=1}^{s} \gamma_t (x_{k,t}^{out})^2 \sum_j (x_{j,t}^{in})^2 + 2\sum_{t=1}^{s} \gamma_t x_{k,t}^{out} \sum_j A_{kj} x_{j,t}^{in} \tag{S2.28}$$
$$+ \left(\sum_{t=1}^{s} \gamma_t x_{k,t}^{out} x_{k,t}^{in}\right)^2.$$

Using the orthonormality conditions Eq. (S2.25) - Eq. (S2.26) and the formulation in Eq. (S2.27), the unique contribution in the case of the multi-component estimator in directed networks is obtained

$$UC(s)_k^{tot} = \sum_{t=1}^{s} \gamma_t^2 \left((x_{k,t}^{in})^2 + (x_{k,t}^{out})^2 \right) + \left(\sum_{t=1}^{s} \gamma_t x_{k,t}^{out} x_{k,t}^{in} \right)^2. \tag{S2.29}$$

The unique contribution when accounting separately for the *out* and *in* properties, applying Eq. (S2.10) and Eq. (S2.11), reads

$$\Delta SE^{out} = \sum_j \left[-\left(\sum_{t=1}^{s} \gamma_t x_{k,t}^{out} x_{j,t}^{in} \right)^2 - 2A_{kj} \sum_{t=1}^{s} \gamma_t x_{k,t}^{out} x_{j,t}^{in} \right]$$

and

$$\Delta SE^{in} = \sum_i \left[-\left(\sum_{t=1}^{s} \gamma_t x_{i,t}^{out} x_{k,t}^{in} \right)^2 + 2A_{ik} \sum_{t=1}^{s} \gamma_t x_{i,t}^{out} x_{k,t}^{in} \right].$$

Going through some algebra and applying the definition in Eq. (S1.8), one has

$$UC(s)_k^{out} = \frac{1}{TSS} \sum_{t=1}^{s} \gamma_t^2 (x_{k,t}^{out})^2, \tag{S2.30}$$

and

$$UC(s)_k^{in} = \frac{1}{TSS} \sum_{t=1}^{s} \gamma_t^2 (x_{k,t}^{in})^2. \tag{S2.31}$$

S2.5 Estimation results

We tested our framework on 36 networks freely available on the *Suite Sparse Matrix Collection*[11]. Our analysis includes all of the binary asymmetric matrices collected in the database, sized $N \leq 2000$. Other networks included in the analysis are (as named as in the database):

- Pajek/Kohonen - size $N = 4470$;
- Rajat/rajat01 - size $N = 6833$;
- SNAP/p2p-Gnutella09 - size $N = 8114$;
- Gleich/wb-cs-stanford - size $N = 9914$;
- SNAP/p2p-Gnutella04 - size $N = 10879$.

The results obtained from our tests are shown in Figure S1.

The values of adjusted coefficient of determination, R_a^2 are higher than those shown in Figure 1, which were obtained from the application of our framework to undirected networks. This is mainly due to the fact that we are using two properties to characterise each node. As a consequence, the estimators (see Table 2) applied in case of directed networks project the information of the adjacency matrix from N^2 to $2N$, reducing the information gap. Also for directed networks, the one-component estimators perform poorly with respect to the two-component estimator. The hub-authority algorithm, however, has better performances than the degree, in particular when considering larger networks.

References

1. Everett, M. & Borgatti, S. Induced, endogenous and exogenous centrality. *Soc. Networks* **32**, 339–344 (2010).
2. Newman, M. E. *Network - An introduction* (Oxford University Press, 2010).
3. Borgatti, S. & Everett, M. Models of core/periphery structures. *Soc. networks* **21**, 375–395 (2000).

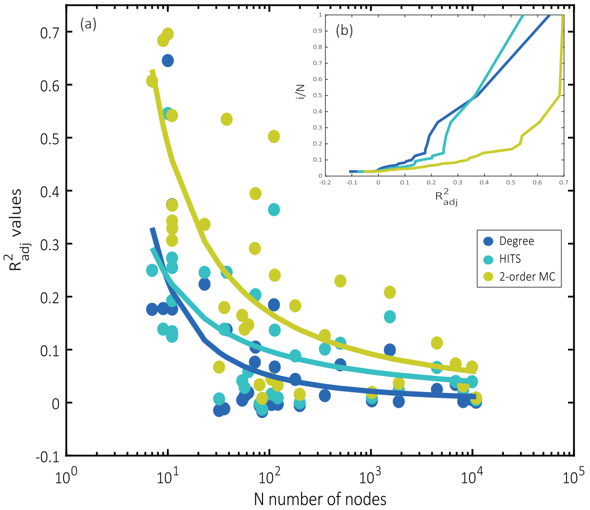

Figure S1. **(a)** Values of the coefficient of determination R_a^2 in semi-log scale obtained through the centrality-based estimators degree, hub-authority and multi-component (MC). Each dot refer to a directed network in the *Sparse Matrix* database[11]. Power-law curves are fitted to the data to facilitate visual comparison. **(b)** Cumulative frequency curves for the R_a^2 obtained by the three estimators.

4. Bonacich, P. Factoring and weighting approaches to status scores and clique identification. *J. Math. Sociol.* **2**, 113–120 (1972).

5. Bonacich, P. Power and Centrality: A Family of Measures. *Am. J. Sociol.* **92**, 1170–1182 (1987).

6. Rencher, A. *Methods of Multivariate Analysis* (John Wiley & Sons, Inc., 2002).

7. Katz, L. A new status index derived from sociometric analysis. *Psychometrika* **18** (1953).

8. Golub, G. H. & Van Loan, C. F. *Matrix computations*, vol. 3 (JHU Press, 2012).

9. Eckart, C. & Young, G. The approximation of one matrix by another of lower rank. *Psychometrika* **1**, 211–218 (1936).

10. Skillicorn, D. *Understanding complex datasets: data mining with matrix decompositions* (CRC press, 2007).

11. Davis, T. A. & Hu, Y. The University of Florida sparse matrix collection. *ACM Transactions on Math. Softw. (TOMS)* **38**, 1 (2011).

12. Kaplan, W. & Lewis, D. *Calculus and Linear Algebra: Vector spaces, many-variable calculus, and differential equations*, vol. 2 (John Wiley & Sons, 1970).

13. Kleinberg, J. M. Authoritative sources in a hyperlinked environment. *J. ACM* **46**, 604–632 (1999).

Acknowledgements

The authors acknowledge ERC funding from the *CWASI* project (ERC-2014-CoG, project 647473).

Author contributions statement

C.S., G.C., F.L. and L.R. conceived and designed the study. C.S. performed the experiments. C.S., G.C., F.L. and L.R. analysed the data. C.S. wrote the manuscript and made the figures for the results. G.C., F.L., and L.R. edited the manuscript. All authors reviewed the manuscript.

Additional information

Competing interests: The authors declare no competing interests, neither financial nor non-financial.
Data availability: The dataset used to perform this research is freely available on-line at *the SuiteSparse Matrix Collection*[11] https://sparse.tamu.edu/. The authors are willing to provide further details upon request.
